# A Panel of Ancestry Informative Markers for the Complex Five-Way Admixed South African Coloured Population

**DOI:** 10.1371/journal.pone.0082224

**Published:** 2013-12-20

**Authors:** Michelle Daya, Lize van der Merwe, Ushma Galal, Marlo Möller, Muneeb Salie, Emile R. Chimusa, Joshua M. Galanter, Paul D. van Helden, Brenna M. Henn, Chris R. Gignoux, Eileen Hoal

**Affiliations:** 1 Molecular Biology and Human Genetics, MRC Centre for Molecular and Cellular Biology and the DST/NRF Centre of Excellence for Biomedical TB Research, Faculty of Medicine and Health Sciences, Stellenbosch University, Tygerberg, South Africa; 2 Biostatistics Unit, Medical Research Council, Tygerberg, South Africa; 3 Statistics Department, University of Western Cape, Cape Town, South Africa; 4 Computational Biology Group, Department of Clinical Laboratory Sciences, Institute of Infectious Disease and Molecular Medicine, University of Cape Town, Medical School, Cape Town, South Africa; 5 University of California San Francisco, San Francisco, California, United States of America; 6 Department of Ecology and Evolution, Stony Brook University, Stony Brook, New York, United States of America; Universitat Pompeu Fabra, Spain

## Abstract

Admixture is a well known confounder in genetic association studies. If genome-wide data is not available, as would be the case for candidate gene studies, ancestry informative markers (AIMs) are required in order to adjust for admixture. The predominant population group in the Western Cape, South Africa, is the admixed group known as the South African Coloured (SAC). A small set of AIMs that is optimized to distinguish between the five source populations of this population (African San, African non-San, European, South Asian, and East Asian) will enable researchers to cost-effectively reduce false-positive findings resulting from ignoring admixture in genetic association studies of the population. Using genome-wide data to find SNPs with large allele frequency differences between the source populations of the SAC, as quantified by Rosenberg et. al's 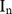-statistic, we developed a panel of AIMs by experimenting with various selection strategies. Subsets of different sizes were evaluated by measuring the correlation between ancestry proportions estimated by each AIM subset with ancestry proportions estimated using genome-wide data. We show that a panel of 96 AIMs can be used to assess ancestry proportions and to adjust for the confounding effect of the complex five-way admixture that occurred in the South African Coloured population.

## Introduction

The predominant population group in the Western Cape, South Africa, is the admixed group known as the South African Coloured (SAC). The SAC had their origins in the diverse groups in the early days of Cape history, including European settlers from 1652, the slaves they brought in from Indonesia, India and other parts of Africa, local Bantu-speakers, and the indigenous Khoe-San. They therefore constitute a complex combination of continental populations [1]. Genetic variation between humans can be ascribed to differences between individuals within populations (85–90%) and to differences between populations (10–15%) [2]–[5]. As humans migrated out of Africa, genetic drift or adaptation resulted in different frequencies of genetic variants in the resultant populations. It is often possible to cluster individuals into population groups that correspond to their self-reported ancestry because of these differences [6]. Admixture occurs when two or more previously separated population groups produce offspring, and it is a well-known confounder in genetic association studies [7]–[9]. In case-control genetic studies, if cases have a different proportion of ancestry from a source population compared to controls, associations found may be related to ancestry rather than disease [10]. It is therefore important to incorporate ancestry in regression models used in genetic association studies of admixed populations. Given genome-wide markers for individuals from an admixed population, principal components or ancestry proportions estimated by solving a multinomial model can be used as covariates to adjust for admixture. However, obtaining genome-wide markers in small follow-up or candidate gene association studies may be prohibitively expensive. Ancestry informative markers (AIMs) are those polymorphisms with the greatest difference in frequency between populations. AIMs can be used as a cost-effective alternative to genome-wide data, if the markers have different allele frequencies in the source populations of the admixed population.

Panels of AIMs have been drawn up for specific populations and purposes. Kosoy et al. set out to find AIMs to determine continental origin and admixture proportions for populations common in America [11]. A list of 128 SNPs were produced by considering the effect of a SNP for distinguishing ancestry independently of the contribution of other SNPs in the data set. This list was later reduced to 93 SNPs [12]. To distinguish between three populations, Galanter et al. [13] used the locus specific branch length (LSBL) of a SNP statistic measured between each pair of three populations [14]. The LSBL was calculated per SNP to develop a panel of AIMs for a diverse set of admixed populations in the Americas that has African, European and Native American ancestry. These AIMs are equally informative for each of the source ancestries, and the panel was shown to provide accurate ancestry proportion estimates by comparing with robust estimates inferred from genome-wide data. SNPs may also be selected by evaluating their combined effect using a performance function. Lao et al. [15] used an asymptotic approximation of the 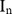-statistic calculated for multiple markers as a performance function [16], [17]. Lao showed that only ten SNPs are required to distinguish the continental ancestry of non-admixed individuals from Eurasia, Africa, America and East Asia. Paschou et al. selected SNPs with the highest loadings summed across the top principal components [18]. This study found that 14 SNPs can differentiate continental ancestry, 100 SNPs differentiate the intra-continental ancestry of the Chinese and Japanese populations, and 200 AIMs were necessary for the admixed Puerto Rican population.

A number of studies showed that the SAC received genetic contributions from click-speaking Africans (African San), Bantu-speaking Africans (African non-San), European, South and East Asians [1], [19]–[22]. The large cohort of SAC individuals used in this paper represents the same population used in the genome-wide analysis performed by De Wit et al. [1] and Chimusa et al. [19]. De Wit et al. found that the cohort received large proportions of ancestry from African San, African non-San and European populations, and a smaller proportion of Asian ancestry. The Asian ancestry was most closely related to a Gujarati Indian population, followed by low levels of ancestry from East Asia. Similar proportions of ancestry were found by Quantana-Murci et al. [22] and Chimusa et al. [19]. These findings are consistent with historical records. Men outnumbered women in the early Cape Society and mixed liaisons were common [1], [23]–[26]. The establishment of mission stations from the mid 1700s onwards further facilitated the integration of European, African (particularly Xhosa) and Khoe-San ancestries [1], [25], [27]. A large proportion of imported slaves originated from Bengal [1], [23]. Bengalis are genetically similar to the Gujarati Indians [20] used to represent the South Asian component in the De Wit and Chimusa studies. The small East Asian ancestry component may be ascribed to the “free black” Chinese who formed 9% of the Cape Town population in the early 1800s [1], [23], [25], [27]. This is more plausible than Indonesian ancestry, since the majority of the cohort are not Muslim and therefore unlikely to form part of the group known as the Cape Malay [1].

Sets of AIMs published by a number of studies [5], [11]–[13], [15], [28]–[30] are not suited to the SAC, since the Khoe-San was not considered as a separate population, or an insufficient number of Khoe-San individuals were used. Complex admixture models such as the five-way admixture that occurred in the SAC, with different levels of genetic distance between source populations, were also not considered. We therefore developed a panel of AIMs tailored to the SAC and assessed its accuracy compared to genome-wide data. Although all the methods discussed above select markers that are informative of ancestry, we also set out to ensure that the selected marker set is reasonably small and as efficient as possible in predicting ancestry. Preliminary investigations indicated that the method introduced by Galanter et al. [13] had the greatest chance of success, and we therefore adapted this method to allow more than three source populations.

## Materials and Methods

Our first step in selecting AIMs was to obtain genome-wide data from populations that are representative of the founding groups of the SAC. Using this data and various different methods to select AIMs, we then set out to find SNPs where the allele frequencies are the most differentiated between the various source populations.

Since the purpose of the AIMs is to adjust for the effects of admixture in genetic studies of the SAC, we assessed the accuracy of various candidate AIM panels by measuring the correlation between ancestry proportions estimated for a large study group of admixed individuals using AIMs and proportions estimated using genome-wide data. We used this information to select a final panel of AIMs of reasonable size.

Finally, we assessed whether the selected panel can be applied to four small South African Coloured study groups from different geographical locations, by measuring the correlation between AIM and genome-wide estimated ancestry proportions.

### Ethics Statement

Approval from the Ethics Committee of the Faculty of Health Sciences, Stellenbosch University (project registration numbers 95/072 and NO6/07/132), was obtained for the Cape Town study group presented in this study. Blood samples for DNA were collected with written informed consent. Sampling and DNA consent from the ‡ Khomani San and individuals who self-identified as “Coloured” in Upington, South Africa and neighboring villages occurred in 2011 and 2012. Institutional Review Board (IRB) approval was obtained from Stanford University and Stellenbosch University (project registration number N11/07/210). ‡ Khomani N|u-speaking individuals, local community leaders, traditional leaders, non-profit organizations and a legal counselor were all consulted regarding the aims of this research, prior to collection of DNA, and regular feedback was given to the community. This research was conducted according to the principles expressed in the Declaration of Helsinki.

### Data

Genome-wide data were obtained from a large study group of individuals who self-identified as South African Coloured and who resided in the Cape Town suburbs of Ravensmead and Uitsig. DNA samples collected from the study group were genotyped on the Affymetrix GeneChip Human Mapping 500K Array Set. More details regarding the sampling and study site are described by [1]. After SNP calling, SNPs that failed a missing threshold of 5%, a minor allele frequency threshold of 1% or a HWE test with an alpha level of 0.0001 were removed. Outliers, related individuals and individuals with a genotyping rate of less than 95% were then removed, resulting in a data set of 733 individuals.

Genome-wide data of four small admixed study groups from different geographical locations were obtained as follows. The first group came from a ‡ Khomani San community in the region of Upington in the Northern Cape, where DNA samples were collected from 21 unrelated individuals who either self-identified as Coloured or had at least one parent who self-identified as Coloured. The samples were genotyped on the Illumina 550K and Illumina OmniExpress (700K) platforms. SNPs that failed a missing threshold of 5% and a minor allele frequency threshold of 0.5% were removed from the data set. Data published by Schlebusch et al. [31] was used for the remaining groups. This data includes three admixed study groups of 20 individuals each. Two of the study groups comprise Coloured individuals from Colesberg in the Northern Cape and Wellington in the Western Cape, respectively. The third study group comprises 20 individuals from the community known as the Karretjie people in the Colesberg region. High proportions of Khoe-San ancestry are present in the Karretjie people [31], and it is thought that they also have European and Bantu ancestry. The DNA samples were genotyped on the Illumina Omni 2.5M SNP chip. The non-imputed data set was used, and no additional SNP quality control steps were performed.

The populations described in Table S1 of Chimusa et al. [19] were considered as potential source populations for the SAC. Principal component and ancestry proportion analysis were used to identify populations with relatively high levels of admixture (see Figures S3, S4, S5, S6 of Chimusa et al.), thereby ensuring that only non-admixed source populations were used for AIM selection. Consequently some of the southern and eastern African populations were excluded from subsequent analysis. Individuals in the Khoe-San data sets that showed relatively high levels of admixture were also removed. The HGDP Melanesian and Papua-New Guinean populations were additionally considered as potential source populations in order to have a comprehensive list, but were excluded since the populations did not appear to be closely related to the Cape Town study group (see Figure S1), which fits with the historical evidence. The Khoe-San data set used to represent the Ju|'hoansi population was obtained from a private data access committee (contact corresponding author). The data set represents the same group analyzed by Schlebusch et al. [31], but was genotyped on the Affymetrix genotyping platform instead of the OmniExpress platform, which overlaps better with SNPs in the other source population data sets that were considered.

Chimusa developed a novel algorithm that identifies the best populations to use as proxy source populations for a multi-way admixed population. This algorithm, as described by Bensmail [32], was used to guide selection of the best populations from the candidate proxy source populations identified by the preliminary investigation. The algorithm leverages the idea that LD is created between genetic loci when admixture occurs between previously isolated populations. A score statistic is calculated per candidate reference population, by measuring the correlation between the LD in the admixed population and the allele frequency difference between the candidate reference population paired with another reference population, for all such possible pairs. The results of the algorithm are summarized in Table S1. The top scoring groups per source population were then used to represent the source populations of the SAC. Ideally only the top one or two scoring populations should be selected as reference populations, but this would have resulted in small sample sizes for the African San and African non-San data sets. Consequently all the African San and the top 8 African non-San populations were selected. The Pakistan South Asian population was not used as we did not have historical evidence to support the use of this population. The HapMap CHB Chinese was also excluded since the group appeared to be very similar to the HapMap CHD Chinese. The final source population data set is summarized in Table 1. Figure S2 is a map representing the geographic locations of the source populations of the SAC used in this study, as well as the admixed SAC study groups.

**Table 1:** Source population data.

Source population	Group	Description	Source	Platform	Size
African San (san)	kho	‡ Khomani San from Northern Cape, South Africa	Henn 2011	Illumina 550K	14
	bus	Juu San from South Namibia	Henn 2011	Illumina 650K & 1M	9
	khs	Ju|'hoansi San from North Namibia	Private	Affymetrix 6.0	22
African non-San (afr)	brong	Ghana	Henn 2011	Affymetrix 500K	8
	kongo	Atlantic coast of Congo	Henn 2011	Affymetrix 500K	9
	igbo	Southeastern Nigeria	Henn 2011	Affymetrix 500K	15
	fang	Equatorial Guinea	Henn 2011	Affymetrix 500K	15
	bulala	Central Chad	Henn 2011	Affymetrix 500K	15
	mada	West Cameroon	Henn 2011	Affymetrix 500K	12
	hausa	West Nigeria	Henn 2011	Affymetrix 500K	12
	bamoun	West Cameroon	Henn 2011	Affymetrix 500K	18
European (eur)	CEU	Utah residents with Northern and Western European ancestry, USA	HapMap3	Release 3	111
	TSI	Italians from Italy	HapMap3	Release 3	102
South Asian (sas)	GIH	Gujarati Indians from Houston, Texas, USA	HapMap3	Release 3	97
East Asian (eas)	CHD	Chinese Metropolitan Denver, Colorado, USA	HapMap3	Release 3	106
	JPT	Japanese from Tokyo, Japan	HapMap3	Release 3	113

Data sets used to represent the five source populations of the South African Coloured population. The sample size reflects the group size after relative pairs have been removed. Henn et al. [52] merged the Juu San data from the Human Genome Diversity Project (HGDP) and Schuster et al. [53] and the African non-San data from Bryc et al [54].

AIMs were selected from the set of SNPs found in all of the source population data sets and the Cape Town study group data set. When estimating ancestry proportions of an admixed study group using genome-wide data, SNPs that were not found in all of the source population data sets were first removed, after which SNPs were filtered according to a linkage disequilibrium (LD) threshold. This was done as increased LD found in admixed populations may bias ancestry proportion estimation. Table S2 presents information on the thresholds applied and number of SNPs used for genome-wide ancestry proportion estimation.

### Selecting Ancestry Informative Markers

Rosenberg's 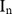-statistic [16] is a measure of the informativeness of a genetic marker in determining an individual's ancestry, for any number of potential source populations. It is often used to select AIMs, as markers with large allele frequency differences between populations will also have a large 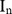-statistic. Galanter et al. selected SNPs based on the LSBL of this statistic, such that the total LSBL calculated for each of the source populations of admixed Latin Americans are equivalent [13].

The LSBL can however only be calculated for three populations and could therefore not be applied to the five source populations of the SAC. We therefore modified their approach to first select a proportion of SNPs according to the 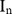-statistic calculated across all of the source populations, and to then select additional SNPs by balancing the total 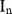-statistic between all pairs of source populations, as described below.

Rosenberg's 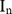-statistic is defined as follows. For a SNP with alleles 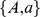 let 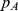 be the frequency of allele 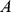 calculated across all the individuals and let 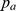 be the frequency of allele 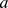 across all the individuals, for that marker. Let 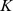 be the number of populations represented by the individuals. Let 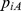 be the frequency of allele 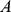 in population 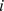 and let 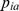 be the frequency of allele 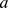 in population 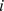. The informativeness of assignment of a SNP is given by
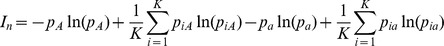
where 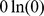 is defined as 0.

It is similar to a log-likelihood ratio, where the ratio is the likelihood that an allele is assigned to one of the populations (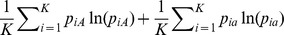), versus the likelihood that the allele is assigned to the average population (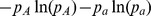).

The allele frequency of each SNP in the data set was calculated, for each source population, and for the population groups included in a source population (for example the East Asian source population comprises the HapMap Japanese and Chinese study groups). SNPs were discarded if they were heterogeneous in these subgroups, based on a Chi-squared test that has a null hypothesis of equal allele frequencies in the subgroups. SNPs were then selected according to the 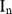-statistic calculated across all the source populations, and the 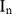-statistic calculated between pairs of populations. Checks were performed before a SNP was accepted as an AIM, to determine whether the SNP was already in the list of AIMs, or was in linkage disequilibrium with any of the SNPs in the list (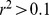), or was located close to any of the SNPs (measured in number of base pairs).

SNPs were selected as follows. The 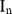-statistic was calculated for all SNPs, across all the source populations, and used to select SNPs with the highest values. This multiple population 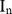-statistic may however be skewed towards populations that are more differentiated (i.e. SNPs from less differentiated populations will contribute less to the statistic and will therefore have a smaller probability of being selected as an informative marker). Additional SNPs were therefore selected by calculating the 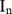-statistic of each SNP for each pair of populations, and then selecting SNPs by balancing the total pairwise 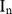
- statistic. For example, for five source populations there are 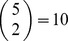 pairs of populations. The pair with the smallest total 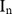-statistic was identified (initially, the total of all pairs are set to zero and are therefore tied) and the SNP with the highest 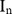-statistic for the identified pair was selected as an AIM. In the case of a tie(s), the SNP with the highest 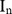-statistic for the tied pair(s) was selected. If the SNP was accepted, its 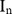-statistic value for the relevant pair was added to the pair's total 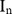-statistic. This process was repeated until the required number of AIMs were accepted.

We generated panels of AIMs of sizes 25, 50, 75,…, 500 using this approach, and experimented with including versus excluding SNPs that are heterogeneous in the populations that constitute a source population, different minimum distances between SNPs and selecting different proportions of markers (0, 0.1, 0.25, 0.5 and 1) using the multiple population 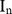-statistic. We also experimented with selecting markers using the implementations provided by Lao et al. [15] and Paschou et al. [18].

### Assessing Ancestry Informative Marker Panels

Let 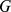 be a matrix of genotypes for each of the 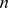 individuals in the data set, 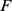 be a matrix of variant allele frequencies for each of the 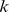 source populations, and 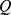 be a matrix of 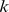 ancestry proportions for each of the 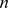 individuals. Ancestry proportions can be estimated by maximizing the likelihood function 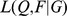.

A strong correlation between ancestry proportions estimated using AIMs for a particular ancestry and ancestry proportions estimated using genome-wide data for the same ancestry would show that the AIMs are informative for that ancestry, even though the number of markers used in the estimation has been much reduced from genome-wide data. We therefore estimated the ancestry proportions of individuals from a combined genome-wide data set composed of both the source population data sets and the Cape Town admixed study group, and identified ancestries as follows. The mean ancestry proportion was calculated for each of the 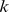 possible ancestries, per source population (using only individuals from that particular source population). The ancestry of a particular source population was then identified by determining which of the 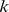 possible ancestries had the largest mean ancestry proportion for that population. The same procedure was used for combined AIM data sets. The correlation between ancestry proportions estimated using the genome-wide data set and proportions estimated using each AIM data set was then calculated per ancestry, using individuals from the admixed study group.

### Software

We modified the Python script provided by Galanter et al. [13] to support more than three source populations. Lao provided us with a Java implementation of his method and we ported the Paschou MATLAB implementation to R [18]. We used PROXYANC to select the best proxy ancestral populations. PLINK [33] was used for quality control filtering, LD filtering and to calculate allele frequencies per population. ADMIXTURE's unsupervised algorithm was used to estimate ancestry proportions [34] and the EIGENSTRAT smartpca program was used for principal component analysis [35]. Statistical analyses were performed using R.

The python script we used to select AIMs can be found in File S1. PROXYANC is found at http://www.cbio.uct.ac.za/proxyanc/software.html.

## Results

The correlation between ancestry proportions estimated using AIMs and proportions estimated using genome-wide data was calculated for AIM sets of increasing size (25, 50,…, 500 SNPs) for different combinations of parameter settings.

For investigating the effect of heterogeneity between subgroups of a source population (the subgroups are summarized under the Population Group heading of Table 1), we used a minimum distance of 100 000 base pairs between SNPs. We selected different proportions of markers using the multiple population 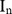-statistic while the remaining SNPs were selected using the pairwise 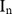-statistic. The difference between the correlation calculated using a AIM set selected from all markers versus the correlation of a AIM set of the same size selected from a marker set containing no heterogeneous SNPs was measured. A positive difference indicates that the AIM set selected from all markers has a higher correlation. Figure S3 depicts the magnitude and direction of the differences measured for the different AIM set sizes and multiple population 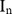-statistic parameter settings. Since 390 of the 400 differences are positive, we ignored heterogeneity in subsequent AIM selections.

Figure S4 shows the differences between correlations estimated using a minimum distance of 100 000 versus a 1 000 000 base pairs between SNPs for different AIM set sizes and multiple population 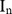-statistic parameter settings. A positive difference indicates that the 100 000 base pair distance has a larger correlation. Although the differences are small and the number of positive differences are not much larger than the number of negative differences, the magnitude of the positive differences are greater compared to the negative differences, except for one of the multiple population 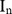-statistic parameter settings. For this reason, we used a minimum distance of a 100 000 base pairs between markers in our subsequent AIM selections.

**Figure 1:**
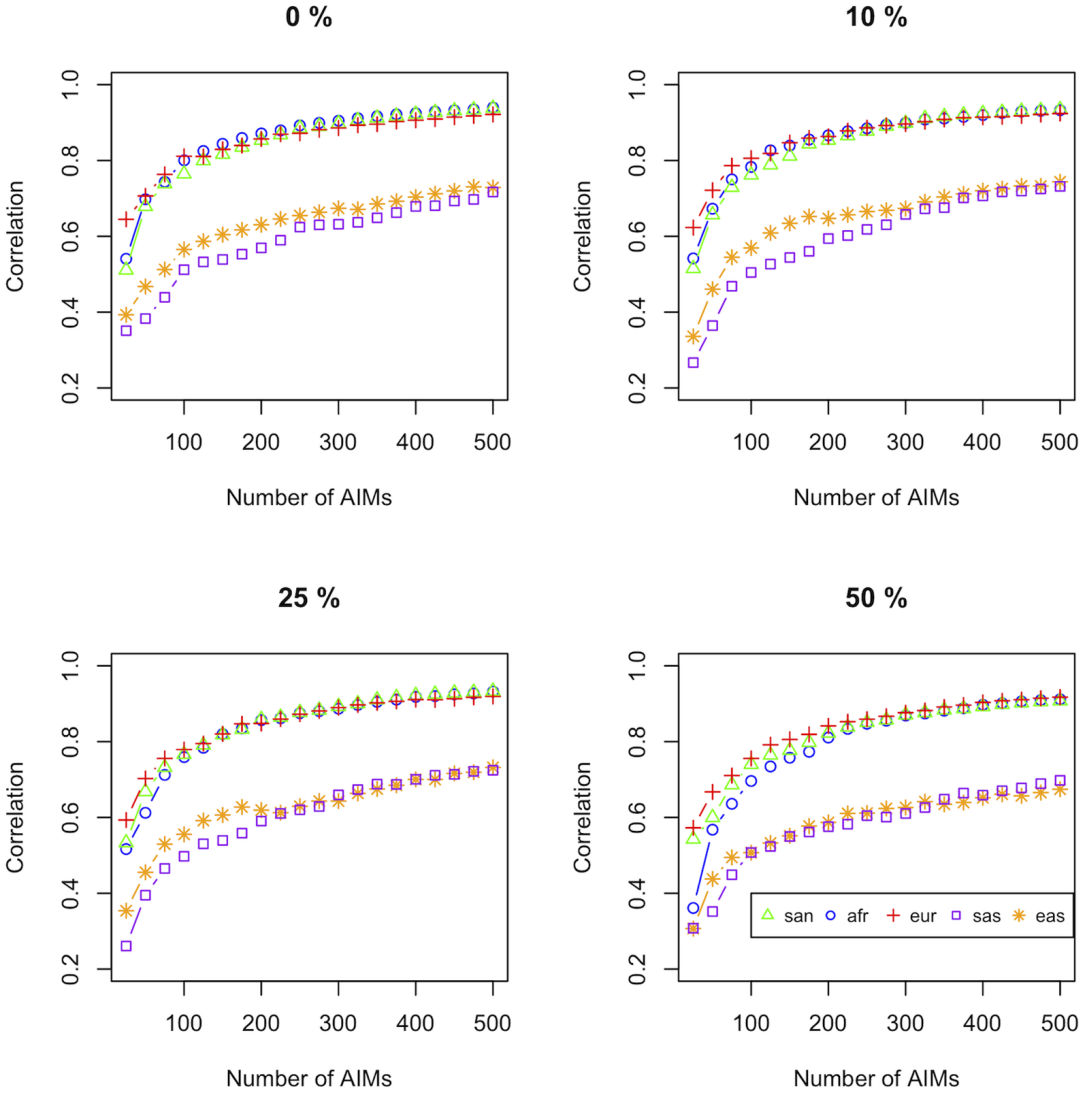
Admixture proportion correlation versus number of AIMs in set. Correlation between admixture proportions estimated using AIMs and proportions estimated using genome-wide data, using AIM sets of increasing size (increments of 25) for the Cape Town study group (n = 733). A proportion of the SNPs in each set of AIMs were selected using the multiple 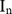-statistic, indicated in each panel as a percentage, while the remaining SNPs were selected using the pairwise 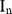-statistic, as described in the Methods section.

A proportion of 0, 0.1, 0.25, 0.5 and 1 markers per set were selected using the multiple population 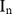-statistic while the remaining SNPs were selected using the pairwise 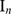-statistic. Selecting all markers using the multiple population statistic (i.e. a proportion of 1) resulted in the ambiguous classification of the source populations for smaller AIM sets; at least 200 SNPs were required for classifying the source populations correctly. Figure 1 shows the correlation per source population for AIM sets of increasing size for the first four multiple population 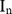-statistic parameter settings. The figure shows that the optimal estimated proportions in terms of cost vs. benefit are obtained using approximately 100 SNPs - incremental improvement in accuracy of estimation using more markers is smaller after this point. Selecting all SNPs by balancing the total pairwise 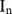-statistic appears to be slightly better compared to selecting some of the SNPs using the multiple population 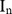-statistic and we therefore used this parameter setting for selecting the final panel of AIMs.

As it is conceivable that future cost reductions may render the cost of genotyping additional SNPs irrelevant, Table S3 presents a panel of 2000 ordered AIMs that were selected using the criteria described above. This large panel can potentially also be used for local ancestry inference. It is currently possible to genotype 96 SNPs cost-effectively on a number of platforms, such as the BeadXpress system, and we therefore evaluated the first 96 SNPs (roughly the optimal number of markers) as our primary panel of AIMs. We also evaluated a panel with 24 additional SNPs, since this slightly larger set of 120 SNPs provides a 3.54% and 5.15% increase in correlation for the estimated African San and South Asian ancestry proportions respectively. This larger marker set can be genotyped using technologies such as Sequenom plexes and Taqman assays, and the results of its evaluation are detailed in the Supporting Information. As expected, for both the 96 and 120 SNP panels the number of AIMs selected per population pair is inversely proportional to the genetic distance between the two populations (Table S4).

Table 2 summarizes the correlation and RSME for the 96 and 120 AIMs. Figure S5 shows Bland Altman plots per ancestral population of the difference between the genome-wide and AIMs estimated proportions versus the genome-wide estimated proportions for each individual (for the 96 AIMs panel). The figure suggests that there are no systematic differences in the ancestry estimation.

**Table 2:** Correlation and RSME of 96 and 120 AIMs.

	96 panel		120 panel	
Ancestry	Correlation	RSME	Correlation	RSME
African San	0.7565	0.0684	0.7905	0.0621
African non-San	0.7930	0.0774	0.8160	0.0719
European	0.8019	0.0554	0.8150	0.0535
South Asian	0.4808	0.0658	0.5283	0.0625
East Asian	0.5665	0.0560	0.5822	0.0522

Correlation and RSME between ancestry proportions estimated using the 96 and 120 AIM panels respectively and proportions estimated using genome-wide data, for the Cape Town study group (n = 733).

As large study groups may require fewer markers to differentiate ancestries [36], the ability of the AIMs to estimate ancestry proportions of a smaller group of South African Coloured individuals were evaluated using permutation testing. 100 individuals were randomly selected from the total of 733 and their ancestry proportions were estimated. The correlation with the genome-wide ancestry proportions for those individuals was then calculated. This process was repeated a 100 times. Figure S6 gives boxplots of the correlation coefficients calculated for each permutation. The red diamonds in the figure are the correlation coefficients calculated using all 733 individuals; this shows that the AIMs perform well for a smaller group of individuals.

**Figure 2:**
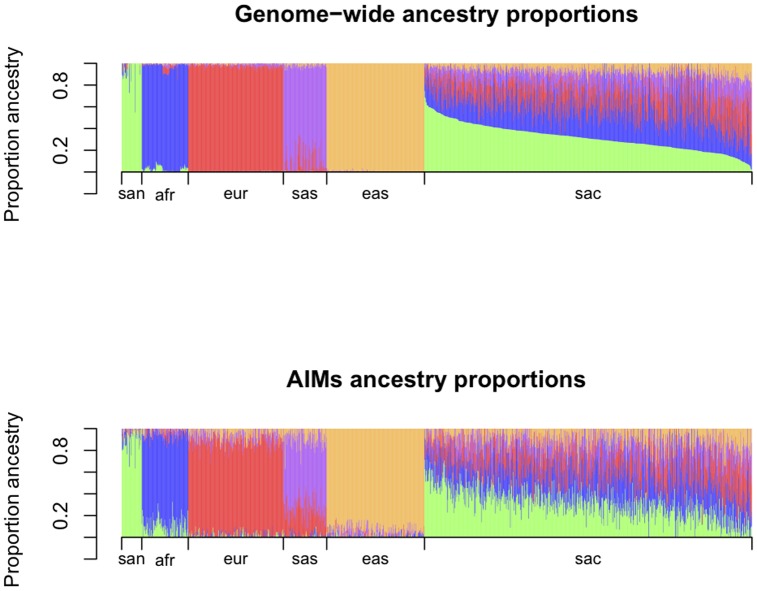
Barplots of ancestry proportions estimated using genome-wide data and using AIMs. In the first panel ancestry proportions were estimated using genome-wide data. The admixed study group (sac) is ordered by proportions of African San, African non-San, European, South Asian and East Asian ancestry. In the second panel ancestry proportions were estimated using 96 AIMs. Individuals appear in the same order as in the first panel.

Markers used to estimate the ancestry proportions of an admixed population can only perform well if they can also distinguish between the source populations of the admixed population. Figure 2 is a barplot of the estimated ancestry proportions for the combined data set, using AIMs and using genome-wide data for the estimation. It shows that for most of the source population individuals, the largest proportion of ancestry is correctly assigned to the relevant population group using AIMs, albeit less well when compared to using genome-wide data. The first three principal components formed using the AIMs for the source population data are depicted in Figure S7, which also suggests that the AIMs can be used to group the five source populations, although the the clusters are wider compared to genome-wide data. Fifty-one percent of the variance in the data is explained by the first three components.

Figure S8 is a histogram of the number of AIMs found on each chromosome, showing that the panel is representative of the entire genome, and that more markers are generally found on the larger chromosomes. This is important since ancestry proportions estimated from markers that are localized to only one part of the genome may differ substantially from an admixed individual's true ancestry proportions across their entire genome. The position of the markers on each chromosome is represented in Figure S9.

**Figure 3:**
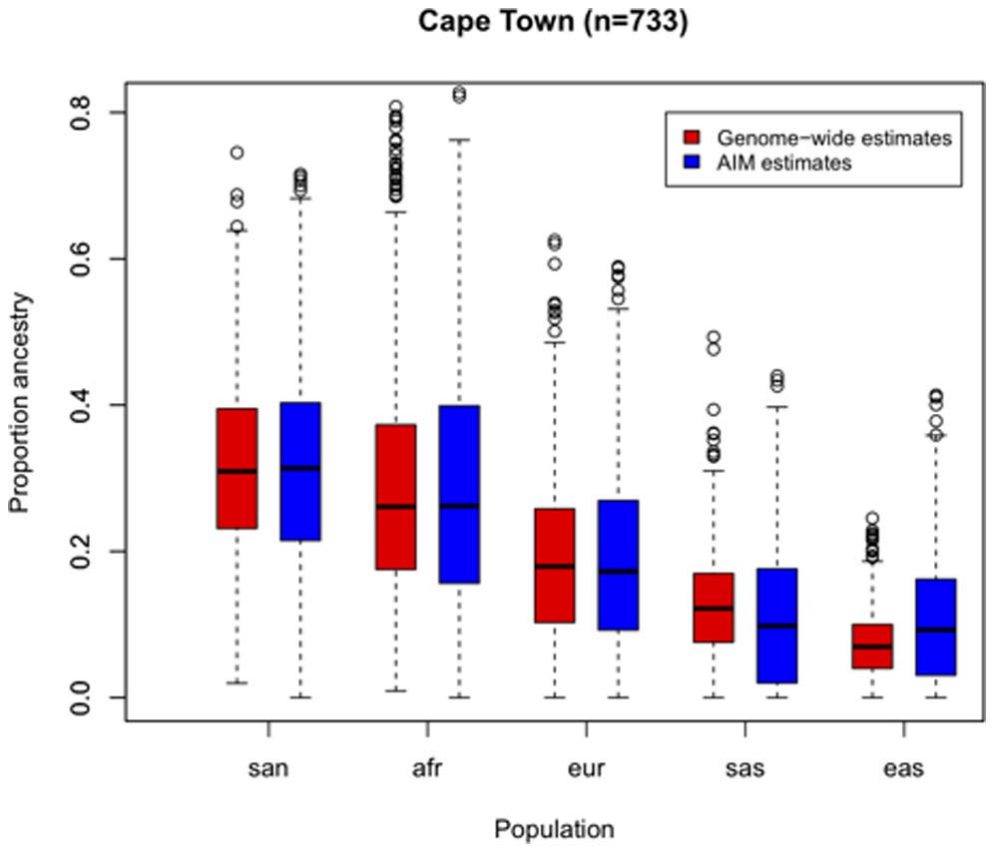
Boxplots of ancestry proportions of the Cape Town study group. Boxplots of ancestry proportions estimated using genome-wide data and proportions estimated using the panel of 96 AIMs are shown in this figure per source population, for the Cape Town study group (n = 733).

Figure 3 depicts boxplots of ancestry proportions estimated using genome-wide data and proportions estimated using AIMs per source population. It shows that the distribution of proportions estimated using AIMs are similar to proportions estimated using genome-wide data, especially for the median ancestry proportions, while the variation of the proportions is only slightly inflated when using AIMs.

To assess the accuracy of the application of the panel to Coloured groups sampled from different geographic locations, we selected markers from the additional Coloured data sets described in Materials and Methods that overlapped with the 120-SNP panel. 76 overlapping SNPs were found in the Upington data set and 84 SNPs were found in the Schlebusch data sets. The number of markers per ancestry pair for each set is shown in Figure S10. Table 3 summarizes the correlations between ancestry proportions estimated using the overlapping AIMs and genome-wide data for each study group. This shows that the markers perform well for each of the groups, considering the reduced size of the AIM panel, possible non-optimal number of markers per ancestry pair and the small group size. Figure S11 depicts boxplots of ancestry proportions estimated using genome-wide data versus proportions estimated using AIMs per source population. The figure illustrates that the distribution of the proportions estimated using AIMs are comparable to the distribution of genome-wide proportions for all the groups. The median and interquartile range of the ancestry proportion estimates inferred from genome-wide data and AIMs are also presented in Table 4, for all the study groups.

**Table 3:** Correlation for different admixed study groups.

Study group	Number AIMs	African San	African non-San	European	South Asian	East Asian
Colesberg (n = 20)	84	0.7661	0.8437	0.8996	0.4675	0.4731
Karretjie (n = 20)	84	0.8436	0.7007	0.7724	0.5590	0.1815
Wellington (n = 20)	84	0.7252	0.7102	0.8008	0.6783	0.3311
Upington (n = 21)	76	0.8747	0.6304	0.8739	0.3777	0.3426

Correlation between ancestry proportions estimated using AIMs and proportions estimated using genome-wide data, for small admixed study groups from different geographic locations. The number of AIMs reflects the number of markers in the 120 panel that were found in the genome-wide data sets of the study groups.

**Table 4:** Ancestry proportion distribution.

Study group	Data set	African San	African non-San	European	South Asian	East Asian
Cape Town	Chip	0.31 (0.23–0.39)	0.26 (0.18–0.37)	0.18 (0.10–0.26)	0.12 (0.08–0.17)	0.07 (0.04–0.10)
(n = 733)	96 AIMs	0.31 (0.21–0.40)	0.26 (0.16–0.40)	0.17 (0.09–0.27)	0.10 (0.02–0.18)	0.09 (0.03–0.16)
	120 AIMs	0.31 (0.22–0.40)	0.27 (0.16–0.39)	0.17 (0.09–0.27)	0.11 (0.03–0.19)	0.08 (0.03–0.15)
Colesberg	Chip	0.33 (0.25–0.40)	0.29 (0.21–0.40)	0.18 (0.10–0.29)	0.05 (0.03–0.09)	0.05 (0.02–0.07)
(n = 20)	84 AIMs	0.31 (0.24–0.35)	0.27 (0.18–0.46)	0.17 (0.03–0.29)	0.07 (0.03–0.19)	0.01 (0.00–0.05)
Karretjie	Chip	0.69 (0.57–0.77)	0.20 (0.15–0.23)	0.08 (0.04–0.12)	0.03 (0.01–0.04)	0.02 (0.01–0.04)
(n = 20)	84 AIMs	0.66 (0.59–0.74)	0.17 (0.08–0.27)	0.04 (0.01–0.16)	0.03 (0.00–0.06)	0.00 (0.00–0.02)
Wellington	Chip	0.13 (0.12–0.15)	0.21 (0.19–0.23)	0.29 (0.24–0.31)	0.17 (0.12–0.23)	0.17 (0.15–0.18)
(n = 20)	84 AIMs	0.14 (0.04–0.25)	0.22 (0.14–0.33)	0.28 (0.19–0.37)	0.10 (0.03–0.16)	0.19 (0.11–0.26)
Upington	Chip	0.61 (0.47–0.72)	0.11 (0.08–0.17)	0.13 (0.10–0.23)	0.04 (0.01–0.09)	0.02 (0.01–0.06)
(n = 21)	76 AIMs	0.62 (0.43–0.67)	0.08 (0.02–0.17)	0.18 (0.07–0.26)	0.02 (0.00–0.07)	0.00 (0.00–0.07)

Median and IQR of the ancestry proportions estimated using genome-wide data and AIMs, per admixed study group.

Tables S5 and S6 present correlations achieved by AIM sets of sizes 88, 194 and 314 AIMs for the Galanter et. al. study [13] and our large SAC study group, as well as sets of 500 and 2000 AIMs for five-way admixture in the SAC. The tables can be used to compare correlations in this study to those obtained by Galanter et al. As expected, the more complex five-way admixture modelling does not yield correlations that are quite as high as the Galanter study for sets of the same size, but this is easily rectified by including additional markers. In addition, when using only the markers that were selected to distinguish the African San, African non-San and European populations and using a simpler three-way admixture model, the correlations are comparable.

We also evaluated AIM panels selected by Lao et al.'s [15] and Paschou et al.'s methods [18], but could not find a smaller set of markers that resulted in stronger correlation between AIM and genome-wide estimated ancestry proportions.

## Discussion

We report the development of a panel of AIMs for the South African Coloured population that enables researchers working with this population to assess population ancestry proportions and correct for substructure. The SAC has a complex history of admixture [1], [22] and has been used in many genetic association studies [37]–[48]. Such candidate gene association studies investigate variants that are often not available in micro-array data. Obtaining genome-wide markers to then simply adjust for admixture may be prohibitively expensive. A viable cost-effective alternative is the genotyping of AIMs. To date, none of the published lists of AIMs have been developed or adequately assessed for distinguishing the ancestries of the SAC, which received genetic contributions from five source populations. Wacholder et al. has argued that confounding due to admixture is minimal for more than three source populations, and that the effect of admixture decrease as the number of strata increases [49]. This study was however limited to U.S. citizens with admixed European ancestry. Studies of multi-way admixed populations formed from different continental populations, that display larger differences in allele frequencies compared to intra-continental populations, may still suffer from the confounding effect of admixture. As an illustration, in a genome-wide tuberculosis (TB) case-control study of the SAC (642 cases and 91 controls), Chimusa et al. found a statistically significant positive correlation between the proportion of African San ancestry and TB susceptibility, and significant negative correlations when regarding European, East Asian and South Asian ancestries [50]. We therefore developed a panel of 96 AIMs for the SAC, by selecting SNPs that can distinguish between all pairs of source populations, as measured by Rosenberg's 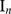-statistic. The AIMs can be used to adjust for the confounding effect of admixture in genetic association studies of the SAC. The correlation between AIMs and genome-wide estimated ancestry proportions may not be sufficient to suggest confidence in ancestry proportions estimated by AIMs at an individual level. However, when the entire study group is considered, the distribution of ancestry proportions are comparable. The panel therefore also has value for inferences about ancestry proportions at the population level. Although we focused on the ability of a small panel of AIMs to adjust for admixture, the entire set of 2000 AIMs can potentially be used to infer local ancestry. Note that accurate local ancestry inference in complex multi-way admixed populations such as the SAC, which has more than three source populations, is currently an unsolved problem. Whilst existing methods may achieve good accuracy on average, inference at particular regions, e.g. regions where the modeled and true ancestral populations differ due to selection, is still problematic.

We have used ancestry proportions estimated using genome-wide data as our gold standard against which to compare proportions estimated using AIMs. However, genome-wide estimated proportions are by no means perfect. Accuracy will vary depending on the choice and number of source populations used. We have therefore taken care to select the best source populations for which genome-wide data is available while taking into account that sample sizes should be reasonable.

Excluding SNPs based on heterogeneity between subgroups of a source population, for example excluding SNPs that are heterogeneous in the three different Khoe-San groups, results in the exclusion of SNPs that can also distinguish source populations. This feature was introduced by Galanter et al. to ensure that their panel of AIMs can be applied to diverse American admixed populations, which may have received genetic contributions from different Native American populations [13]. Since this scenario does not apply to the SAC, and using this criterion results in a lower overall correlation between ancestry proportions estimated using AIMs and proportions estimated using genome-wide data, we ignored heterogeneity between subgroups in our final selection of AIMs.

The ability of the AIMs to distinguish South Asian and East Asian ancestries is markedly lower compared to the African San, African non-San and European ancestries. This could potentially be explained if the groups used as proxies for the South and East Asian source populations are not ideal representations of these ancestries in the SAC, although we have attempted to use the best reference groups for which genome-wide data were available. In addition, the genetic distance between South Asians and Europeans is relatively small compared to the genetic distance between other pairs of populations, and it is therefore more difficult to distinguish. Alternatively, the lower correlation of the Asian ancestries could be ascribed to the small proportions observed in our study groups. In the Galanter et. al. study, ancestry estimates for source populations that contributed less to the admixed population also had a relatively low correlation [13]. Due to these reasons, a much larger panel of AIMs would be required to improve the ability to distinguish the Asian ancestries. As the genetic contribution of the Asian ancestries to the SAC is relatively small, and because South Asians and Europeans are genetically similar, confounding due to the Asian ancestries are likely to be trivial in association studies. The list of AIMs presented in our study does state which source population pair each marker has been selected for. Markers selected for pairs that include the Asian ancestries can therefore easily be excluded, especially when a small panel is required. It is however our opinion that it is important to consider the Asian ancestries, since ignoring them would result in a less accurate overall estimation of ancestry.

The AIMs were selected from a set of markers that were successfully genotyped on the Affymetrix 500K chip for the admixed Cape Town study group, and that overlapped with source population data sets used in this study. The source population data sets were genotyped on a number of different microarray chips, including Illumina chips. It is therefore likely that the markers will also be genotyped successfully by other technologies, such as custom designed genotyping chips, the BeadXpress system, Sequenom plexes and Taqman assays.

According to the 2011 South African census, the majority of individuals who self-identify as South African Coloured reside in the Western Cape province [51]. The Cape Town study group of admixed individuals, recruited from the suburbs of Ravensmead and Uitsig in the Western Cape and who self-identified as South African Coloured, was used to assess the accuracy of the AIMs panel. We therefore believe that our panel of AIMs is applicable to the majority of individuals constituting this population group. We have also shown that the AIMs perform well for other Coloured groups residing in the Western Cape and the Northern Cape. These groups may be genetically distinct from one another due to genetic drift and different dates and levels of admixture between the different source populations. Since we have shown that the AIMs can distinguish the ancestries of the different admixed groups, the panel can also be used to correct for stratification when a study group has not been sampled from a relatively homogeneous admixed population. This is important as recent migration might introduce additional unknown heterogeneity into communities. It remains to be seen how well the AIMs perform in other Southern African mixed ancestry groups, such as the Cape Malay, a group which may have retained some distinction from the general South African Coloured population, groups living in the Eastern Cape and the Basters who reside mainly in Namibia. We have not been able to assess the accuracy of the panel for such groups due to the lack of availability of genome-wide data. It is, however, likely that the AIMs will also be applicable to these groups, since they were formed from the same source populations, or subsets of the same source populations. Consequently, the cost of studies regarding the overall genetic make-up of other Coloured groups can be much reduced. Based on our recent experience in Southern Africa, genotyping 120 AIMs were five times more cost-effective using Sequenom plexes compared to the most cost-efficient micro-array chips, which is particularly relevant when sample sizes are large. This is especially important in the light of limited access to research funding in Southern Africa. Although the cost of micro-array genotyping continues to decline, this also holds true for platforms designed for smaller marker sets, making it difficult to speculate on when the cost reduction will become a moot point.

In summary, we have developed a panel of 96 AIMs that is tailored to the complex five-way admixture that occurred in the South African Coloured population. This panel can be used as a cost effective alternative to genome-wide data for reducing false positive findings resulting from ignoring admixture in genetic association studies of the population.

## Supporting Information

Figure S1 **Ancestry proportion and principal component analysis (PCA) of the SAC and the Oceania HGDP populations.** (A) The proportion of each individual's ancestry. (B) The first and second eigenvectors of the PCA of the combined populations.
(PDF)

Figure S2 **World map with source and admixed populations.** Abbreviations used for the source populations correspond to Table 1. The admixed populations are indicated as follows: Cape Town = cpt, Colesberg = col, Karretjie = kar, Wellington = wel, Upington = upt. The *ceu*, *chd* and *gih* HapMap populations received ancestry from continents that differ from their sampling locations. Their approximate area of origin is in solid colour, with migration shown by arrows.
(PDF)

Figure S3
**Scatter plots of the difference in correlation coefficients against the number of AIMs used in the calculation of the correlations, when ignoring heterogeneity versus removing heterogeneous SNPs.** Both correlations are between ancestry proportions estimated from genome-wide data and ancestry proportions estimated using a set of AIMs selected from the genome-wide data. The difference is between the AIMs selected from all the genome-wide SNPs and those selected from genome-wide SNPs from which markers that are heterogeneous in subgroups of the source populations have been removed. The percentage of SNPs selected using the multiple 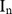-statistic (the remainder were selected using the pairwise 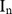-statistic) are shown for each plot. SNPs were selected with a minimum distance of 100 000 base pairs between them.(PDF)

Figure S4
**Scatter plots of the difference in correlation coefficients against the number of AIMs used in the calculation of the correlations, when using a minimum distance of 100 000 base pairs between SNPs versus a 1 000 000 base pairs.** Both correlations are between ancestry proportions estimated from genome-wide data and ancestry proportions estimated using a set of AIMs selected from the genome-wide data. The difference is between the AIMs selected so that there is a minimum distance of 1 000 000 base pairs between them and those selected with a minimum distance of 100 000 base pairs between them. AIM sets were selected from all the genome-wide SNPs. The percentage of SNPs selected using the multiple 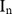-statistic (the remainder were selected using the pairwise 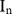-statistic) are shown for each plot.(PDF)

Figure S5 **Bland Altman plots of differences between ancestry proportion estimates.** Bland Altman plots per ancestral population of the difference between the genome-wide and AIMs estimated proportions (y-axis) versus the genome-wide estimated proportions (x-axis) for each individual, using 96 AIMs. Each panel respresents the ancestry proportions of one of the source populations of the SAC.
(PDF)

Figure S6 **Boxplot of permutation correlation.** A boxplot of correlation coefficients calculated in 100 permutations per source population, each permutation comprising a random draw of 100 individuals from the Cape Town study group (n = 733). The correlation was measured between admixture proportions estimated using the panel of 96 AIMs and proportions estimated using genome-wide data. The red diamonds represent the correlation coefficients calculated using the entire study group.
(PDF)

Figure S7 **Principal components formed using genome-wide data and AIMs.** The first two panels show principal components 1 and 2 and 2 and 3 respectively, inferred from the source population genome-wide data. Similarly, panels 3 and 4 shows principal components inferred from 96 AIMs. Each data point represents the score of an individual for a principal component. The legend shows which source population each individual belongs to.
(PDF)

Figure S8 **Histogram of the number of AIMs on each chromosome.** Histogram that represents the number of markers in the panel of 96 AIMs per chromosome.
(PDF)

Figure S9 **Base pair position of AIMs per chromosome.** The figure shows the position in number of base pairs of each of the 96 AIMs per chromosome.
(PDF)

Figure S10 **Number AIMs found in admixed study groups per population pair.** The number of AIMs per source population pair found in the different admixed study group data sets.
(PDF)

Figure S11 **Boxplot of ancestry proportions of small admixed study groups.** The distribution of ancestry proportions estimated using genome-wide data and proportions estimated using AIMs are shown in this figure for the small admixed study groups, per source population. The Colesberg, Karretjie and Wellington study groups are each comprised of 20 individuals and 84 AIMs were used to estimate ancestry proportions. The Upington study group comprises 21 individuals and 76 AIMs were used to estimate ancestry proportions.
(PDF)

Table S1 Proxy ancestry scores. The results of the PROXYANC algorithm ordered by the magnitude of the score, per source population.
(PDF)

Table S2 The number of markers used for genome-wide ancestry proportion estimation per admixed study group. After the set of SNPs that overlap with all the source population data sets was found, a LD filter was applied to each admixed study group, using a window size of 50 SNPs and a shift size of 10 SNPs. Only the remaining SNPs were used for ancestry proportion estimation.
(PDF)

Table S3 2000 AIMs. The top 2000 markers selected by our algorithm as AIMs for the South African Coloured population are found in *table_s3.xls*. The table presents information on the marker location, allele frequency and population pair that a marker was selected for. The list is ordered according to marker selection, i.e. the panel of 96 AIMs evaluated are the first 96 markers in the table.
(XLS)

Table S4 Number markers selected per source population pair. The number of markers selected per pair of source populations, for the panels of 96 and 120 AIMs. The number of markers selected are inversely proportional to the genetic distance between the populations that constitute the pair, as measured by Fst.
(PDF)

Table S5 Correlation obtained by Galanter et al. Correlation between ancestry proportions estimated using 88, 194 and 314 AIMs and proportions estimated using genome-wide data, for two of the admixed study groups in the Galanter et al. study.
(PDF)

Table S6 Correlation obtained in the Cape Town study group for comparision to the Galanter et al. study. Correlation between ancestry proportions estimated using 88, 194 and 314 AIMs and proportions estimated using genome-wide data, for a 5-way and 3-way admixture model. Correlations for AIM sets of sizes 500 and 2000 are also given for the 5-way admixture model.
(PDF)

File S1 **AIM selection script.** A zip file containing the python script we used to select AIMs (AIMs_generator.py), a text file with instructions for running the script, and two example input parameter files.
(ZIP)
