# Supplementary figures and images for: A Panel of Ancestry Informative Markers for the Complex Five-Way Admixed South African Coloured Population
Source: PLoS One. 2013 Dec 20;8(12):e82224. doi: 10.1371/journal.pone.0082224 (PMC3869660; doi:10.1371/journal.pone.0082224)

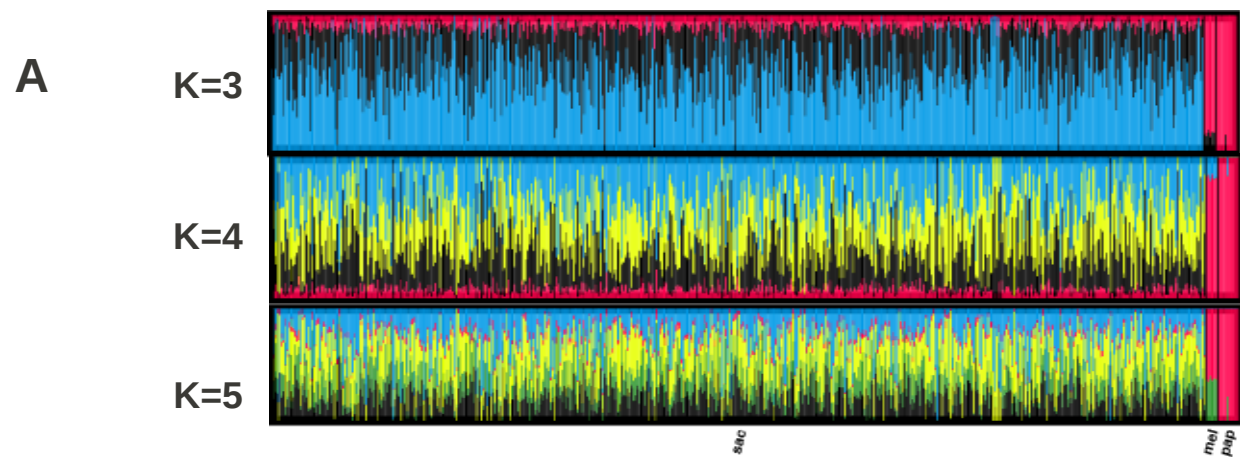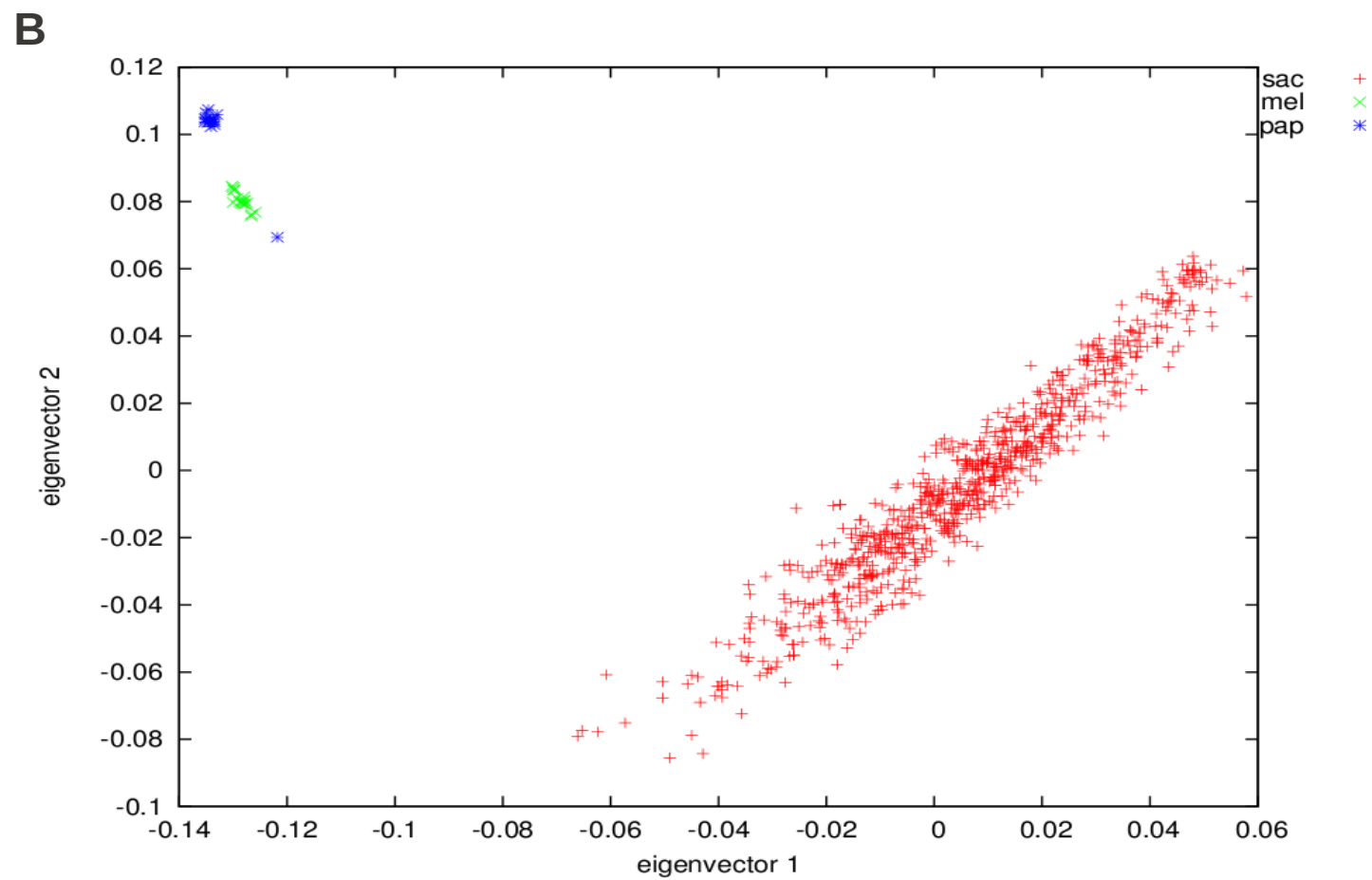

Supplement: Figure S1 — Ancestry proportion and principal component analysis (PCA) of the SAC and the Oceania HGDP populations. (A) The proportion of each individual's ancestry. (B) The first and second eigenvectors of the PCA of the combined populations. (PDF) [file pone.0082224.s001.pdf]

- African San
- African non-San
- European
- South Asian
- East Asian
- SAC

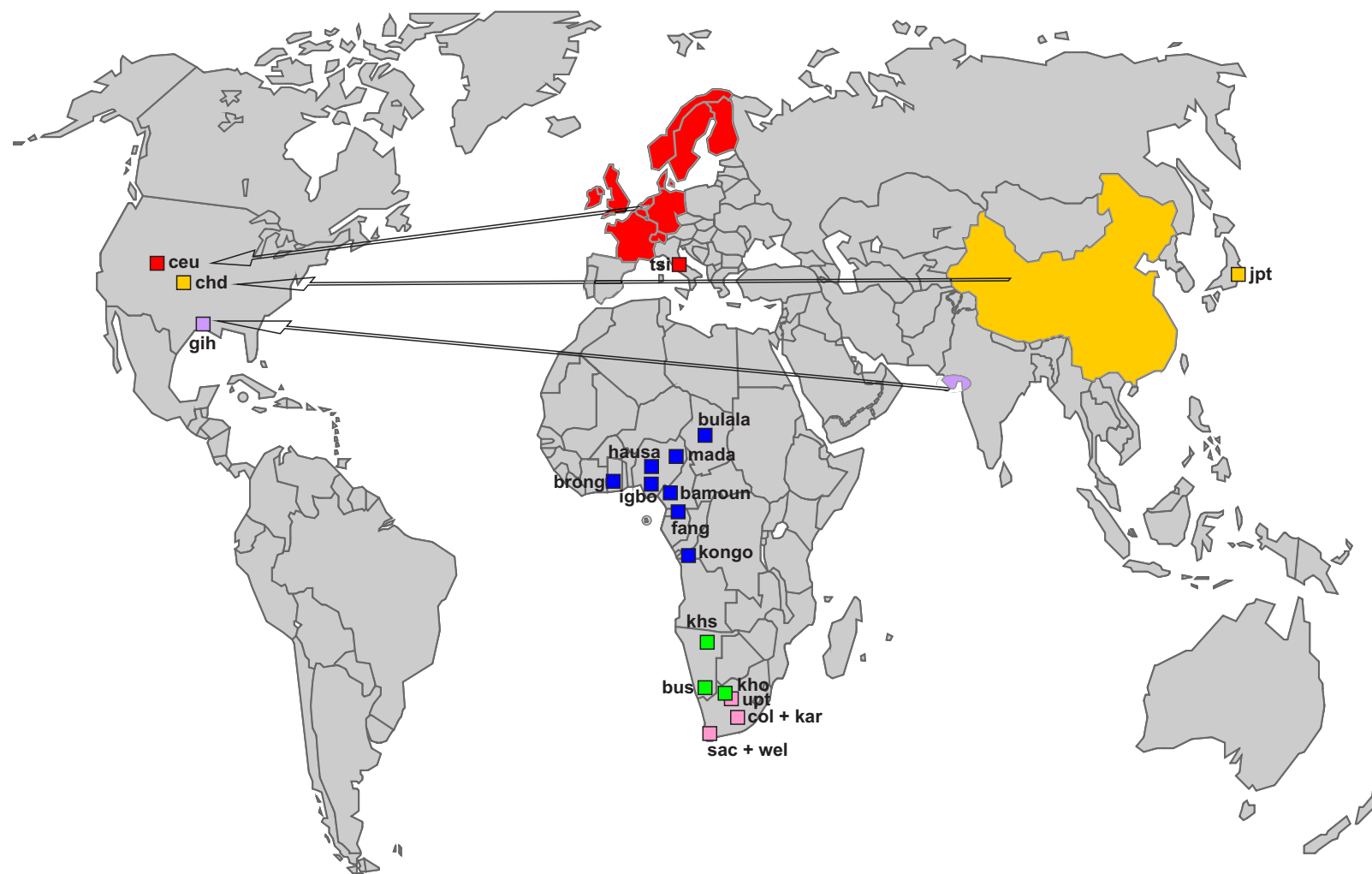

Supplement: Figure S2 — World map with source and admixed populations. Abbreviations used for the source populations correspond to Table 1. The admixed populations are indicated as follows: Cape Town = cpt, Colesberg = col, Karretjie = kar, Wellington = wel, Upington = upt. The ceu, chd and gih HapMap populations received ancestry from continents that differ from their sampling locations. Their approximate area of origin is in solid colour, with migration shown by arrows. (PDF) [file pone.0082224.s002.pdf]

**0 %**

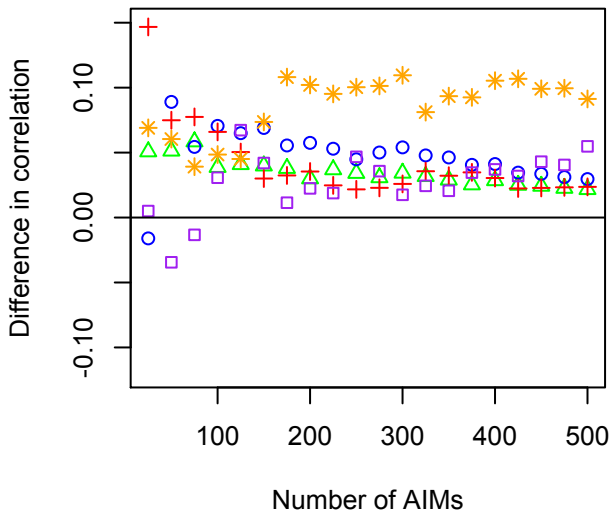

**10 %**

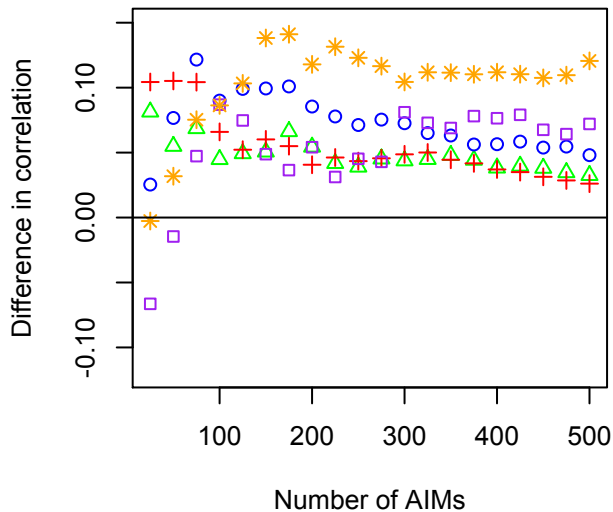

**25 %**

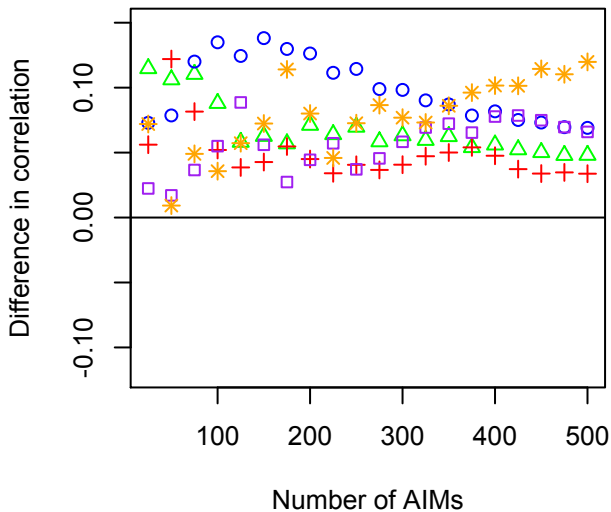

**50 %**

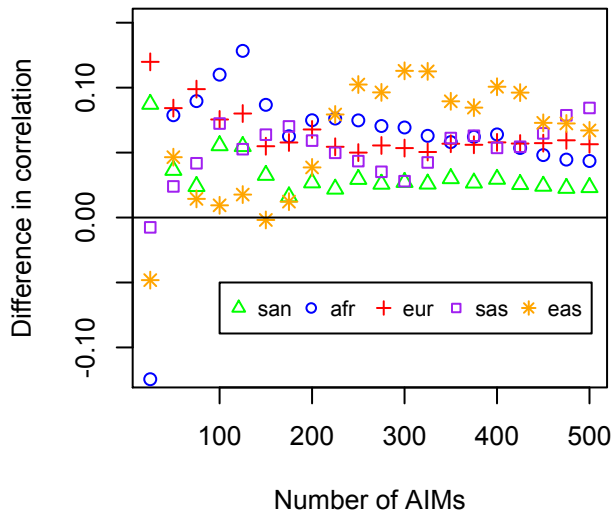

Supplement: Figure S3 — Scatter plots of the difference in correlation coefficients against the number of AIMs used in the calculation of the correlations, when ignoring heterogeneity versus removing heterogeneous SNPs. Both correlations are between ancestry proportions estimated from genome-wide data and ancestry proportions estimated using a set of AIMs selected from the genome-wide data. The difference is between the AIMs selected from all the genome-wide SNPs and those selected from genome-wide SNPs from which markers that are heterogeneous in subgroups of the source populations have been removed. The percentage of SNPs selected using the multiple -statistic (the remainder were selected using the pairwise -statistic) are shown for each plot. SNPs were selected with a minimum distance of 100 000 base pairs between them. (PDF) [file pone.0082224.s003.pdf]

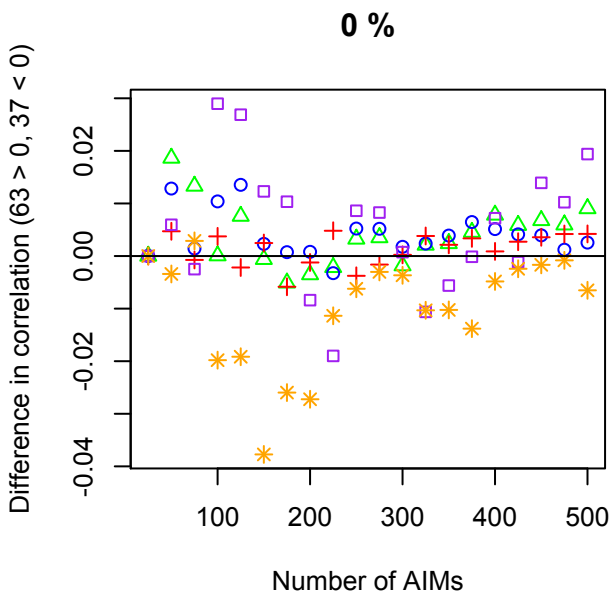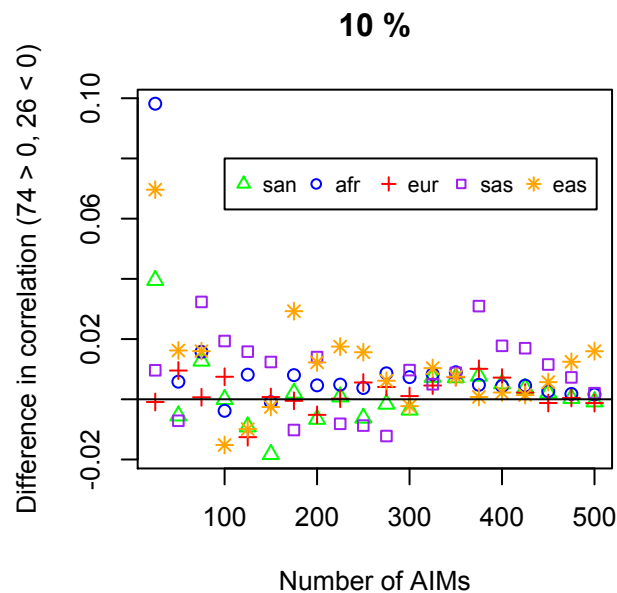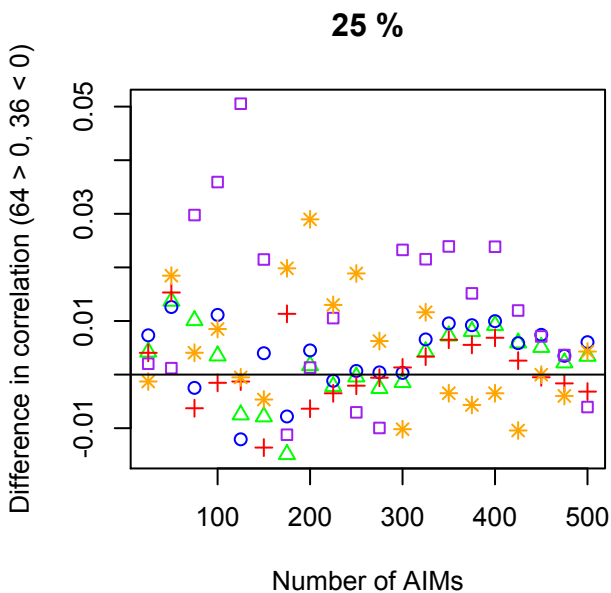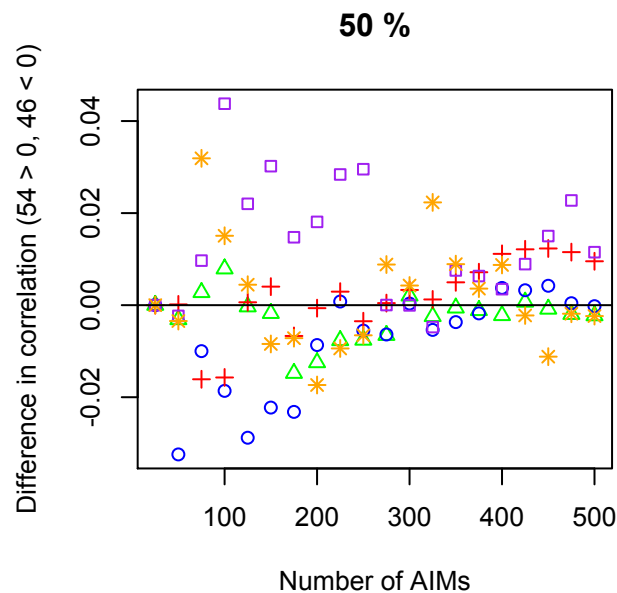

Supplement: Figure S4 — Scatter plots of the difference in correlation coefficients against the number of AIMs used in the calculation of the correlations, when using a minimum distance of 100 000 base pairs between SNPs versus a 1 000 000 base pairs. Both correlations are between ancestry proportions estimated from genome-wide data and ancestry proportions estimated using a set of AIMs selected from the genome-wide data. The difference is between the AIMs selected so that there is a minimum distance of 1 000 000 base pairs between them and those selected with a minimum distance of 100 000 base pairs between them. AIM sets were selected from all the genome-wide SNPs. The percentage of SNPs selected using the multiple -statistic (the remainder were selected using the pairwise -statistic) are shown for each plot. (PDF) [file pone.0082224.s004.pdf]

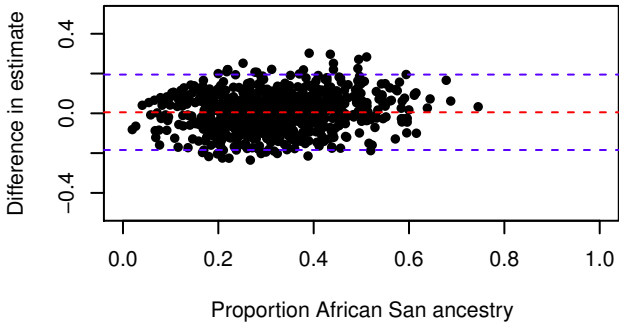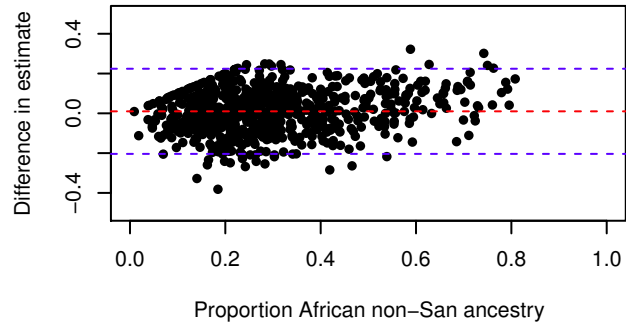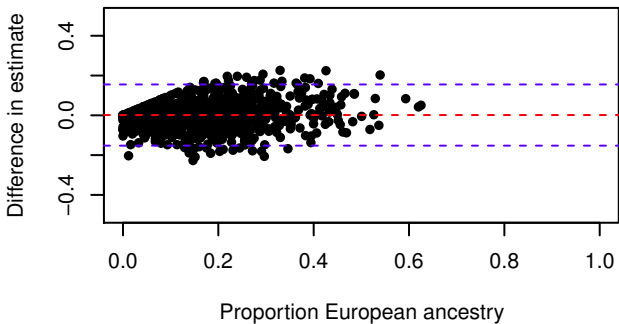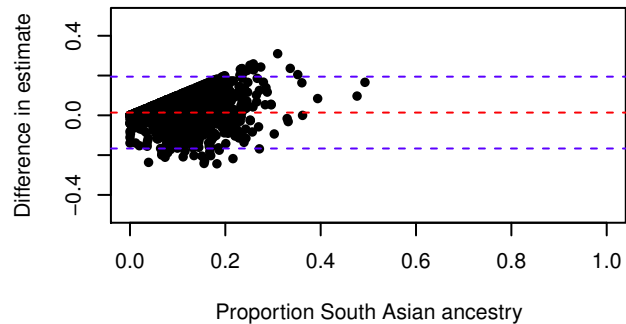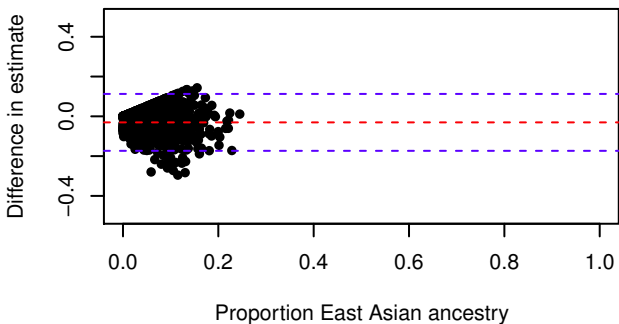

Supplement: Figure S5 — Bland Altman plots of differences between ancestry proportion estimates. Bland Altman plots per ancestral population of the difference between the genome-wide and AIMs estimated proportions (y-axis) versus the genome-wide estimated proportions (x-axis) for each individual, using 96 AIMs. Each panel respresents the ancestry proportions of one of the source populations of the SAC. (PDF) [file pone.0082224.s005.pdf]

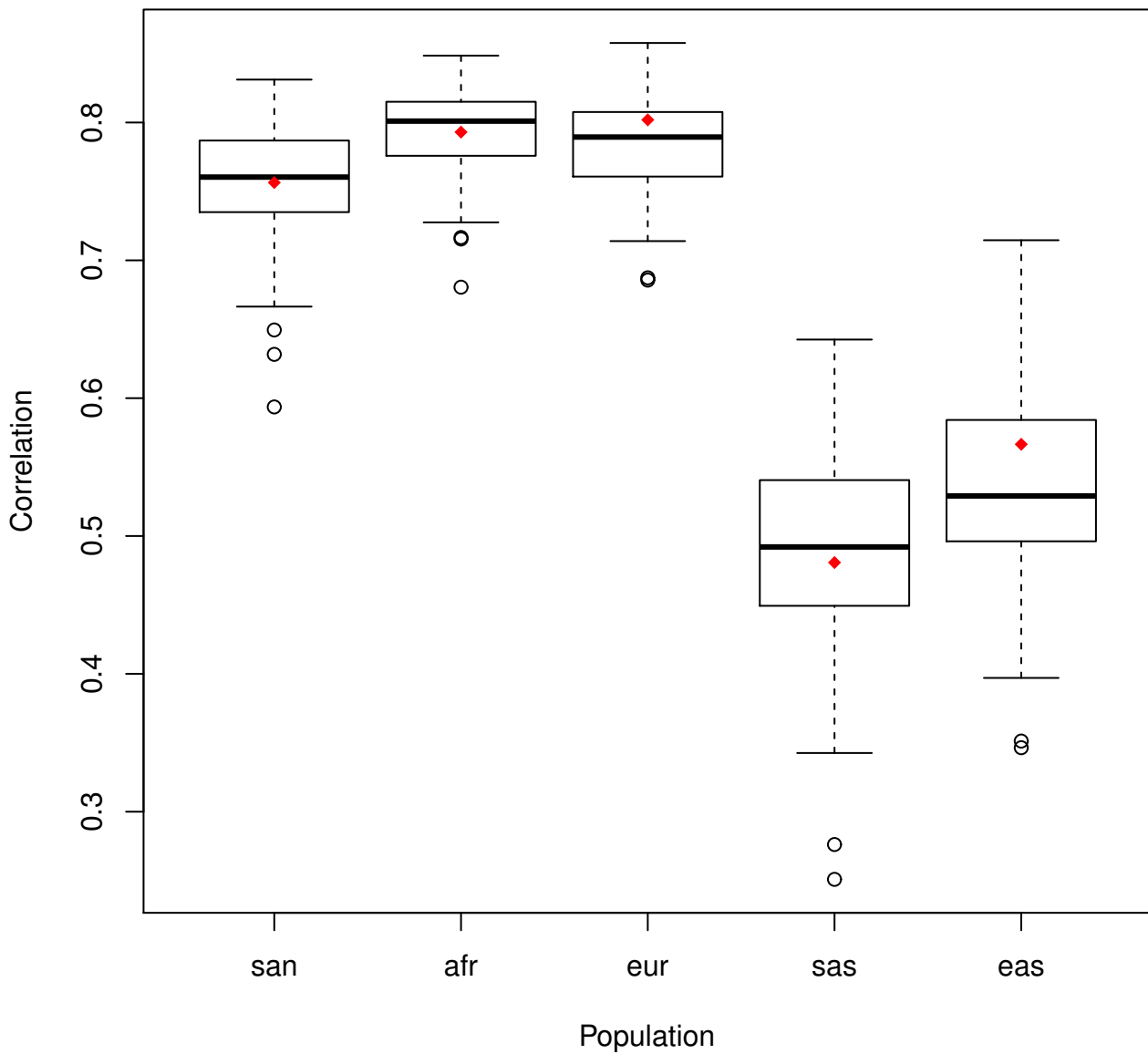

Supplement: Figure S6 — Boxplot of permutation correlation. A boxplot of correlation coefficients calculated in 100 permutations per source population, each permutation comprising a random draw of 100 individuals from the Cape Town study group (n = 733). The correlation was measured between admixture proportions estimated using the panel of 96 AIMs and proportions estimated using genome-wide data. The red diamonds represent the correlation coefficients calculated using the entire study group. (PDF) [file pone.0082224.s006.pdf]

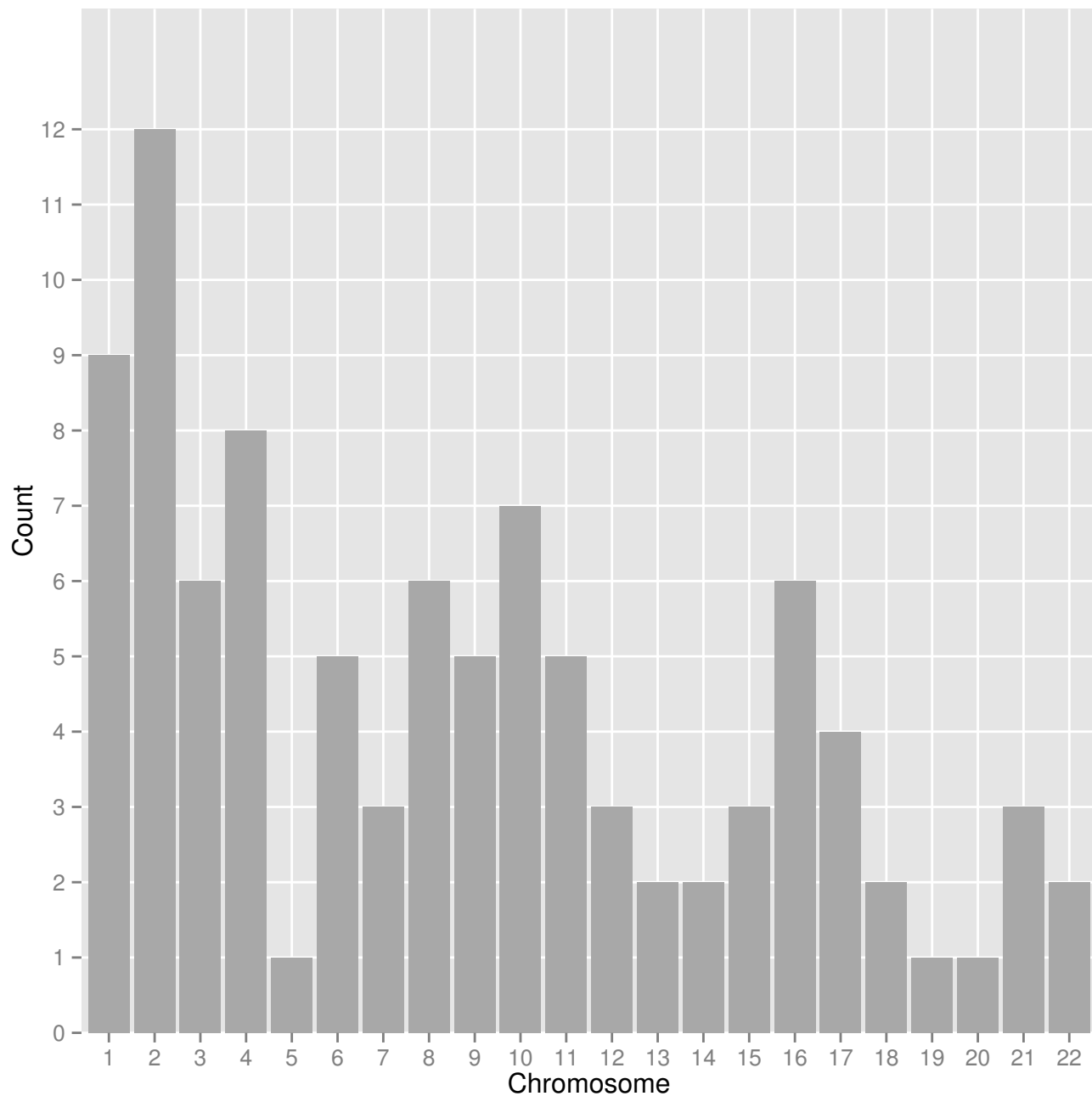

Supplement: Figure S8 — Histogram of the number of AIMs on each chromosome. Histogram that represents the number of markers in the panel of 96 AIMs per chromosome. (PDF) [file pone.0082224.s008.pdf]

Chromosome

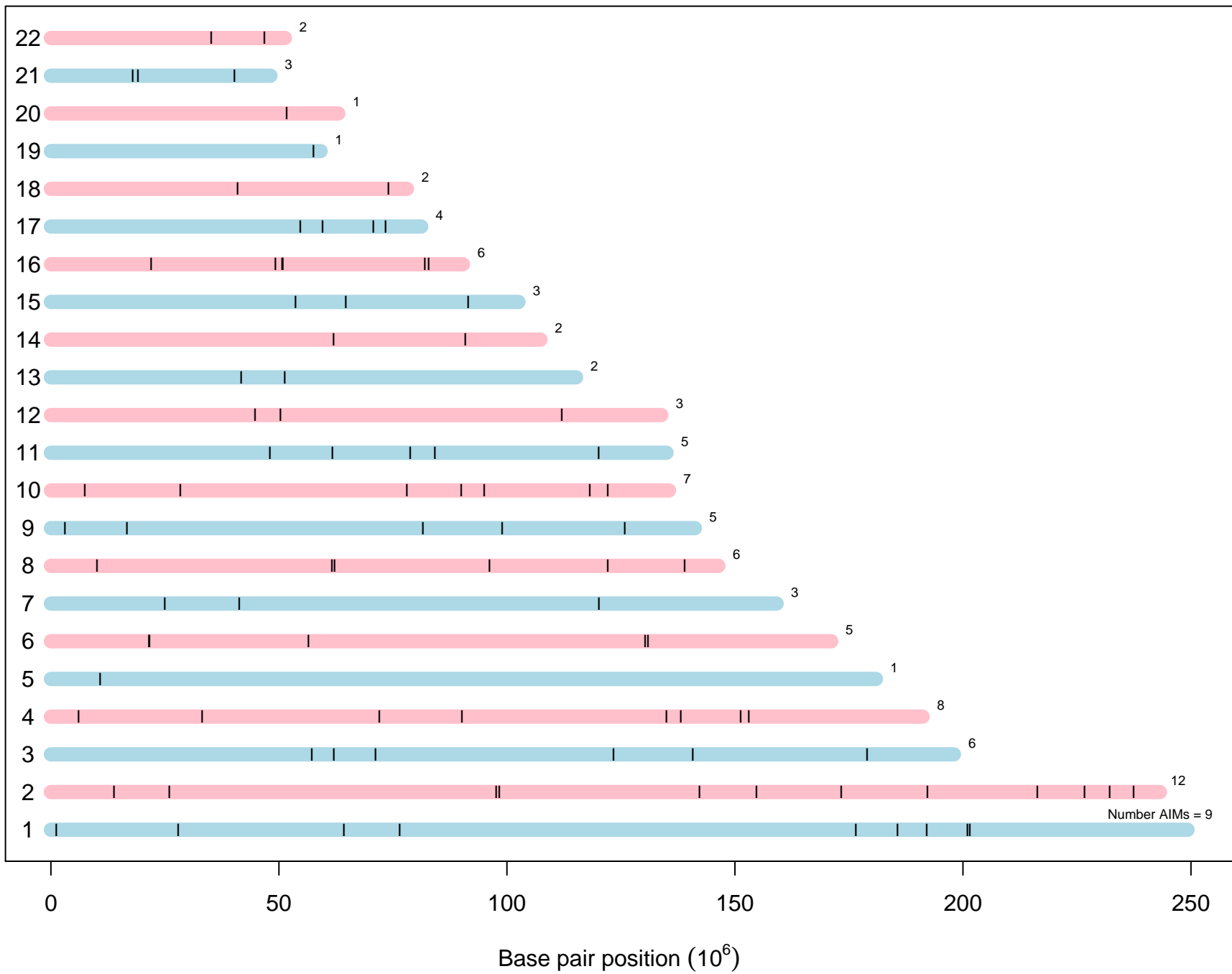

Supplement: Figure S9 — Base pair position of AIMs per chromosome. The figure shows the position in number of base pairs of each of the 96 AIMs per chromosome. (PDF) [file pone.0082224.s009.pdf]

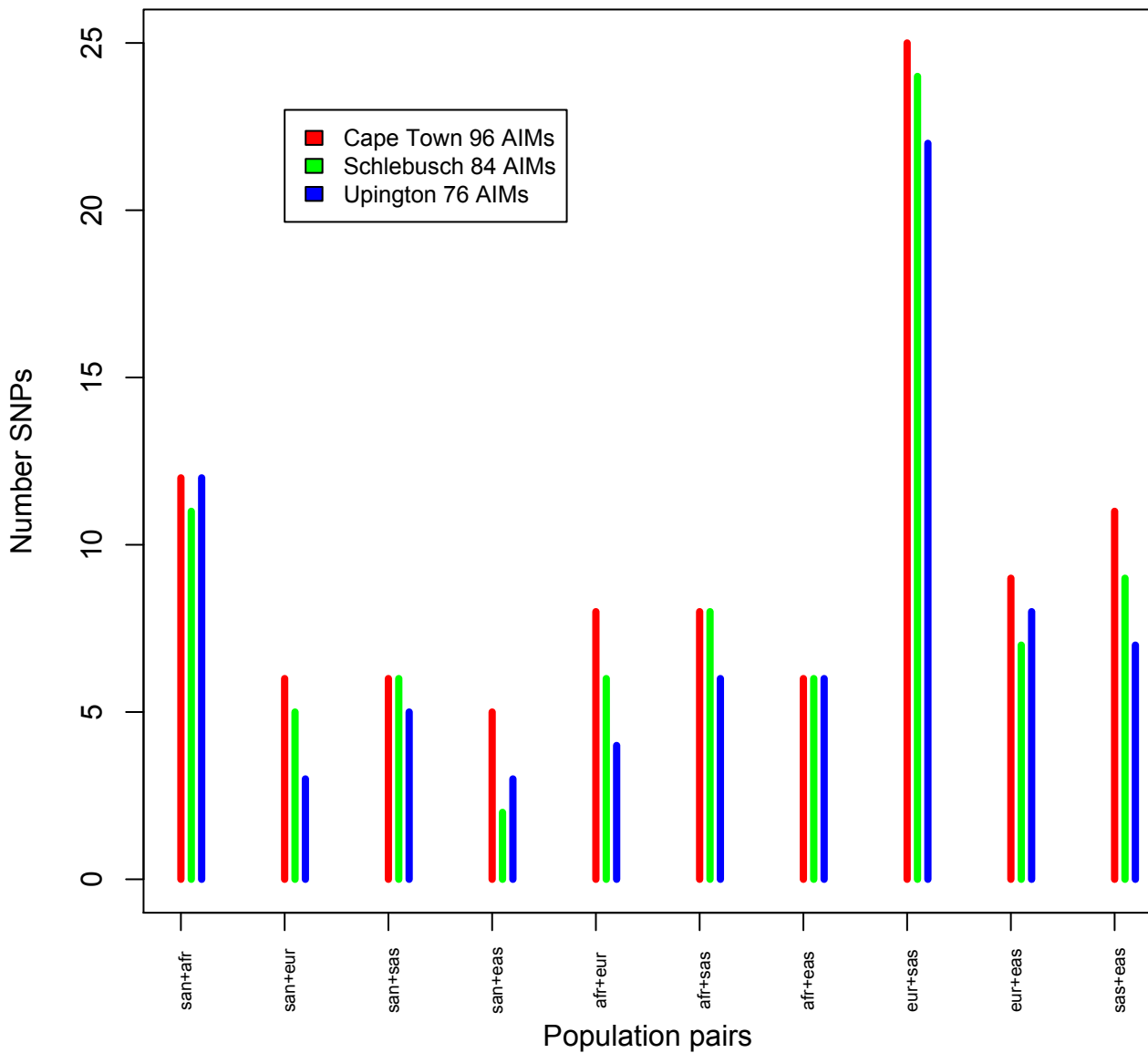

Supplement: Figure S10 — Number AIMs found in admixed study groups per population pair. The number of AIMs per source population pair found in the different admixed study group data sets. (PDF) [file pone.0082224.s010.pdf]

**Colesberg**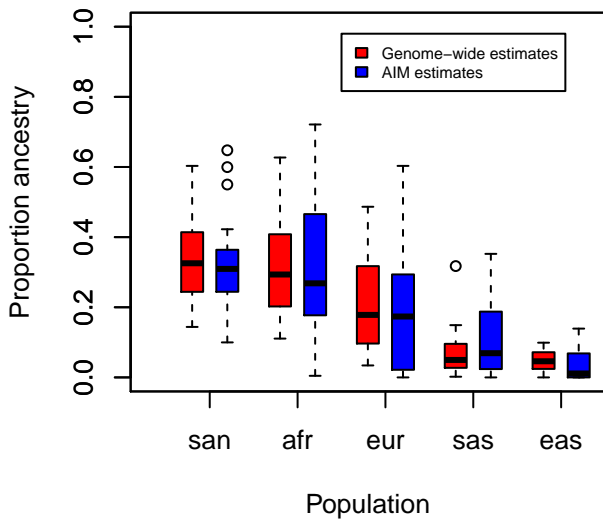**Karretjie**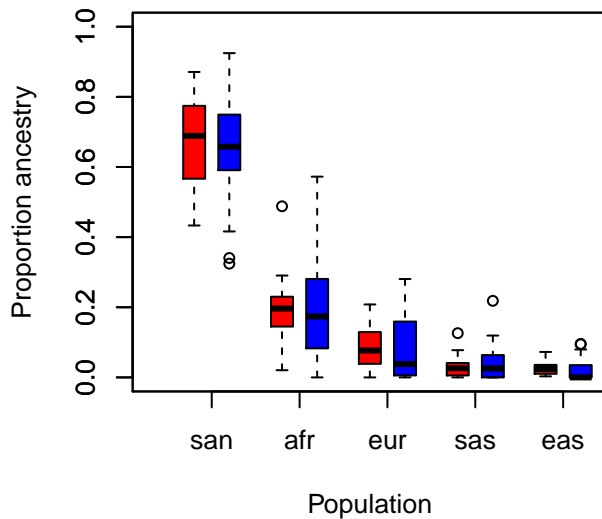**Wellington**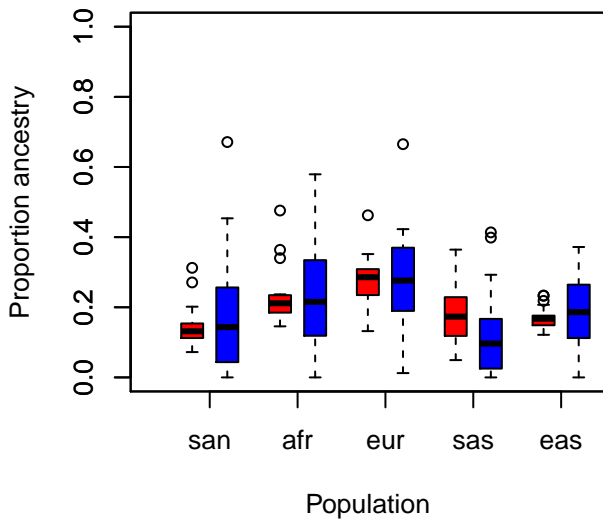**Uppington**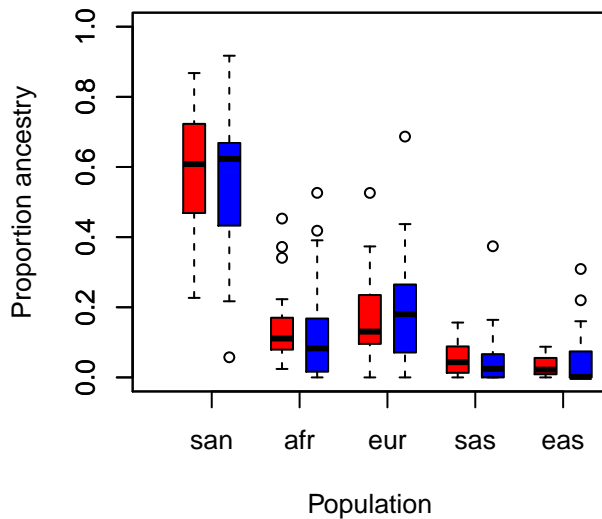

Supplement: Figure S11 — Boxplot of ancestry proportions of small admixed study groups. The distribution of ancestry proportions estimated using genome-wide data and proportions estimated using AIMs are shown in this figure for the small admixed study groups, per source population. The Colesberg, Karretjie and Wellington study groups are each comprised of 20 individuals and 84 AIMs were used to estimate ancestry proportions. The Upington study group comprises 21 individuals and 76 AIMs were used to estimate ancestry proportions. (PDF) [file pone.0082224.s011.pdf]
