# Supplementary material for: A Panel of Ancestry Informative Markers for the Complex Five-Way Admixed South African Coloured Population
Source: PLoS One. 2013 Dec 20;8(12):e82224. doi: 10.1371/journal.pone.0082224 (PMC3869660; doi:10.1371/journal.pone.0082224)
Supplement: Figure S7 — Principal components formed using genome-wide data and AIMs. The first two panels show principal components 1 and 2 and 2 and 3 respectively, inferred from the source population genome-wide data. Similarly, panels 3 and 4 shows principal components inferred from 96 AIMs. Each data point represents the score of an individual for a principal component. The legend shows which source population each individual belongs to. (PDF) [file pone.0082224.s007.pdf]

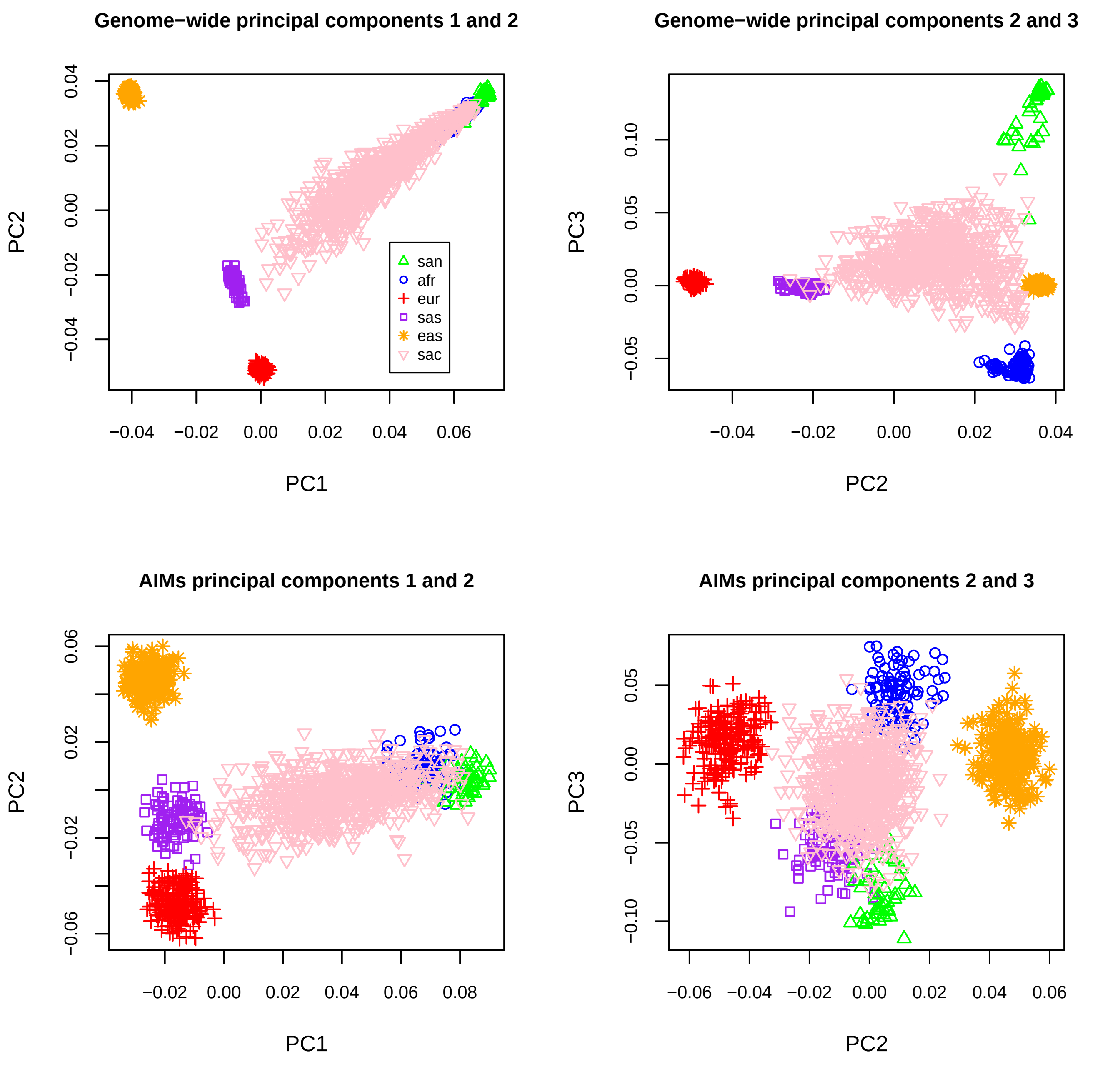

Genome−wide principal components 1 and 2
Genome−wide principal components 2 and 3
AIMs principal components 1 and 2
AIMs principal components 2 and 3
PC1
PC2
PC3
san
afr
eur
sas
eas
sac
