# Supplementary material for: A Panel of Ancestry Informative Markers for the Complex Five-Way Admixed South African Coloured Population
Source: PLoS One. 2013 Dec 20;8(12):e82224. doi: 10.1371/journal.pone.0082224 (PMC3869660; doi:10.1371/journal.pone.0082224)
Supplement: Table S1 — Proxy ancestry scores. The results of the PROXYANC algorithm ordered by the magnitude of the score, per source population. (PDF) [file pone.0082224.s012.pdf]

Table S1: Proxy ancestry scores. The results of the PROXYANC algorithm ordered by the magnitude of the score, per source population.

Candidate proxy	Score	Description	Source
		**African San**	
kho	163	Khoe-San South Africa	Henn 2011
bus	156	Khoe-San South Namibia	Henn 2011
khs	127	Khoe-San Namibia	Private[1]
		**African non-San**	
brong	899	Ghana	Henn 2011
kongo	809	Atlantic coast of Congo	Henn 2011
igbo	807	South Eastern Nigeria	Henn 2011
fang	668	Equatorial Bantu	Henn 2011
bulala	565	Central Chad	Henn 2011
mada	482	Cameroon	Henn 2011
hausa	449	West Africa	Henn 2011
bamoun	438	Cameroon	Henn 2011
yri	186	Yoruba in Ibadan	HapMap3
yor	160	Yoruba in Ibadan	Henn 2011
fulani	118	West-central Africa	Henn 2011
		**European**	
ceu	252	Northern European	HapMap3
tsi	198	Italy	HGDP
fre	165	French-france	HGDP
bas	162	Basque-France	HGDP
rus	154	Russian-russia	HGDP
sar	152	Sardinian-Italy	HGDP
		**South Asian**	
gih	191	Gujarati Indians	HapMap3
han	159	Pathan-Pakistan	HGDP
		**East Asian**	
chd	226	Chinese in Denver	HapMap3
chb	205	Han Chinese in Beijing	HapMap3
jpt	191	Japanese in Tokyo	HapMap3
jap	159	Japanese-Japan	HGDP
han	159	Pathan-Pakistan	HGDP
mia	153	Miao-China	HGDP
she	149	She-China	HGDP
dai	147	Dai-China	HGDP

[1] Private data access committee
