# Supplementary material for: A Panel of Ancestry Informative Markers for the Complex Five-Way Admixed South African Coloured Population
Source: PLoS One. 2013 Dec 20;8(12):e82224. doi: 10.1371/journal.pone.0082224 (PMC3869660; doi:10.1371/journal.pone.0082224)
Supplement: Table S2 — The number of markers used for genome-wide ancestry proportion estimation per admixed study group. After the set of SNPs that overlap with all the source population data sets was found, a LD filter was applied to each admixed study group, using a window size of 50 SNPs and a shift size of 10 SNPs. Only the remaining SNPs were used for ancestry proportion estimation. (PDF) [file pone.0082224.s013.pdf]

Table S2: The number of markers used for genome-wide ancestry proportion estimation per admixed study group. After the set of SNPs that overlap with all the source population data sets were found, a LD filter was applied to each admixed study group, using a window size of 50 SNPs and a shift size of 10 SNPs. Only the remaining SNPs were used for ancestry proportion estimation.

Study group	Number overlapping markers	Number remaining markers	r^2 threshold
Cape Town (n=733)	50 286	33 125	0.1
Colesberg (n=20)	29 914	14 662	0.3
Karretjie (n=20)	29 914	13 883	0.3
Wellington (n=20)	29 914	15 277	0.3
Upington (n=21)	30 466	16 195	0.3
