# Supplementary material for: A Panel of Ancestry Informative Markers for the Complex Five-Way Admixed South African Coloured Population
Source: PLoS One. 2013 Dec 20;8(12):e82224. doi: 10.1371/journal.pone.0082224 (PMC3869660; doi:10.1371/journal.pone.0082224)
Supplement: Table S4 — Number markers selected per source population pair. The number of markers selected per pair of source populations, for the panels of 96 and 120 AIMs. The number of markers selected are inversely proportional to the genetic distance between the populations that constitute the pair, as measured by Fst. (PDF) [file pone.0082224.s015.pdf]

Table S4: Number markers selected per source population pair. The number of markers selected per pair of source populations, for the panels of 96 and 120 AIMs. The number of markers selected are inversely proportional to the genetic distance between the populations that constitute the pair, as measured by Fst.

**Population pair**	**96 panel**	**120 panel**	**Fst** [1]
African San + African non-San	12	15	0.0841
African San + European	6	7	0.1951
African San + South Asian	6	7	0.1901
African San + East Asian	5	7	0.2331
African non-San + East Asian	6	8	0.1749
African non-San + European	8	9	0.1395
African non-San + South Asian	8	10	0.1297
European + South Asian	25	32	0.0338
European + East Asian	9	11	0.1090
South Asian + East Asian	11	14	0.0760

[1]Calculated using genome-wide data listed in table 1 and the R hierfstat package
