# Supplementary material for: A Panel of Ancestry Informative Markers for the Complex Five-Way Admixed South African Coloured Population
Source: PLoS One. 2013 Dec 20;8(12):e82224. doi: 10.1371/journal.pone.0082224 (PMC3869660; doi:10.1371/journal.pone.0082224)
Supplement: Table S5 — Correlation obtained by Galanter et al. Correlation between ancestry proportions estimated using 88, 194 and 314 AIMs and proportions estimated using genome-wide data, for two of the admixed study groups in the Galanter et al. study. (PDF) [file pone.0082224.s016.pdf]

Table S5: Correlation obtained by Galanter et al. Correlation between ancestry proportions estimated using 88, 194 and 314 AIMs and proportions estimated using genome-wide data, for two of the admixed study groups in the Galanter et al. study.

		Correlation		
**Study group**	**Nr AIMs**	**American**	**European**	**African**
Mexico City Mexicans	314	0.985	0.980	0.748
	194	0.975	0.970	0.693
	88	0.960	0.943	0.592
GALA Puerto Ricans	314	0.735	0.943	0.959
	194	0.656	0.922	0.944
	88	0.520	0.849	0.877
