# Supplementary material for: A Panel of Ancestry Informative Markers for the Complex Five-Way Admixed South African Coloured Population
Source: PLoS One. 2013 Dec 20;8(12):e82224. doi: 10.1371/journal.pone.0082224 (PMC3869660; doi:10.1371/journal.pone.0082224)
Supplement: Table S6 — Correlation obtained in the Cape Town study group for comparision to the Galanter et al. study. Correlation between ancestry proportions estimated using 88, 194 and 314 AIMs and proportions estimated using genome-wide data, for a 5-way and 3-way admixture model. Correlations for AIM sets of sizes 500 and 2000 are also given for the 5-way admixture model. (PDF) [file pone.0082224.s017.pdf]

Table S6: Correlation obtained in the Cape Town study group for comparision to the Galanter et al. study. Correlation between ancestry proportions estimated using 88, 194 and 314 AIMs and proportions estimated using genome-wide data, for a 5-way and 3-way admixture model. Correlations for AIM sets of sizes 500 and 2000 is also given for the 5-way admixture model.

		Correlation				
**Model**	**Nr AIMs**	**African San**	**African non-San**	**European**	**South Asian**	**East Asian**
SAC 5-way admixture	2000	0.977	0.979	0.975	0.884	0.893
	500	0.938	0.939	0.922	0.717	0.730
	314	0.902	0.909	0.892	0.637	0.675
	194	0.845	0.866	0.852	0.563	0.631
	88	0.751	0.779	0.785	0.467	0.563
SAC 3-way admixture	314	0.937	0.944	0.975	-	-
	194	0.916	0.921	0.961	-	-
	88	0.858	0.863	0.930	-	-
